# Validation of the mRNA epitranscriptome: SCARPET reveals that mapped m^1^A sites are inosine

**DOI:** 10.1038/s44319-026-00840-2

**Published:** 2026-06-23

**Authors:** Rohit Nalavade, Aashiq H Mirza, Samie R Jaffrey

**Affiliations:** Department of Pharmacology, Weill Cornell Medicine, Cornell University, New York, NY USA

**Keywords:** Methods & Resources, RNA Biology

## Abstract

A major goal in epitranscriptomics is to define the diversity of modified nucleotides in mRNA. Most candidate sites are discovered using transcriptome-wide mapping methods that rely on antibody enrichment or modification-induced misincorporations during cDNA synthesis. However, these maps are rarely verified biochemically, and in some cases have yielded conflicting conclusions, most notably for *N*^1^-methyladenosine (m^1^A). Initial transcriptome-wide maps reported thousands of internal m^1^A sites while later studies disputed these findings and showed that these sites lacked the expected m^1^A-induced misincorporations. More recently, an evolved reverse transcriptase that detects m^1^A was developed to resolve this issue and was used to map sites that again suggested that internal m^1^A might be widespread. These divergent results underscore the need for biochemical testing of proposed sites. Here, we describe protocols for SCARPET (site-specific cleavage and radiolabeling followed by purification, exonuclease digestion, and thin-layer chromatography) to rapidly and efficiently detect modified nucleotides in RNA. Using SCARPET, we show that newly mapped internal m^1^A sites are not m^1^A, but instead contain inosine from A-to-I editing. However, *N*⁷-methylguanosine (m^7^G) sites, which are also controversial, were confirmed to occur at high stoichiometry. These results confirm that view that m^1^A is extremely rare and establish SCARPET as a robust strategy for validating putative modification sites.

## Synopsis

The reported thousands of internal m¹A and m⁷G sites in mammalian mRNA are controversial. SCARPET along with BioCapT shows that putative m¹A sites instead contain inosine arising from A-to-I RNA editing, while internal m⁷G sites do occur at high stoichiometry.

- SCARPET reliably detects and quantifies diverse RNA modifications.
- Internal m⁷G occurs at considerable stoichiometry in mRNA.
- Putative internal m¹A sites in mRNA are inosine.

## Abstract (web summary)

The reported thousands of internal m¹A and m⁷G sites in mammalian mRNA are controversial. SCARPET along with BioCapT shows that putative m¹A sites instead contain inosine arising from A-to-I RNA editing, while internal m⁷G sites do occur at high stoichiometry.

## Graphical abstract

**Figure:**
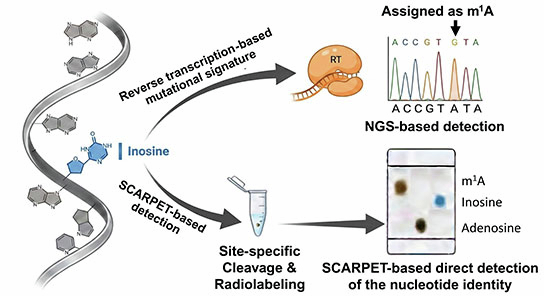

**Figure:**
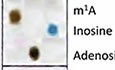

## Introduction

Post-transcriptional chemical modifications of RNA expand the functional complexity of the transcriptome (Cappannini et al, 2023; Gilbert and Nachtergaele, 2023). The first such modification discovered was *N*^6^-methyladenosine (m^6^A), identified in the 1970s (Canaani et al, 1979; Desrosiers et al, 1974; Perry and Kelley, 1974), with early evidence implicating m^6^A in the destabilization of mRNA (Sommer et al, 1978). A major advance occurred in 2012 with the development of the first transcriptome-wide m^6^A mapping methods (Dominissini et al, 2012; Meyer et al, 2012), which accelerated the field by revealing that m^6^A is not randomly distributed, but instead is enriched in defined regions of mRNA, preferentially localized in certain transcripts, and dynamically regulated across cellular states. These mapping efforts also laid the groundwork for functional studies that have uncovered key roles for m^6^A in diverse biological processes (Gilbert and Nachtergaele, 2023; Murakami and Jaffrey, 2022).

Given the well-established functions of m^6^A, extensive efforts have been devoted to identifying other chemically modified nucleotides within mRNA (Sun et al, 2023). Unlike m^6^A, which is abundant enough to be detected using relatively crude techniques such as paper chromatography in the 1970s (Canaani et al, 1979), most other mRNA modifications are present at much lower levels and thus require more sensitive detection approaches, including mass spectrometry (Herbert et al, 2024; Kong et al, 2023). Low abundance of an RNA modification poses a significant challenge for its transcriptome-wide mapping, where the scarcity of true modification sites increases the relative contribution of background noise, thereby elevating false positive rates (Grozhik and Jaffrey, 2018; Wiener and Schwartz, 2021). Consequently, biochemical validation of mapped sites, at single-nucleotide resolution, can help to ensure that a mapped site reflects a modification rather than a background signal.

Several RNA modifications were initially reported to be widespread in mRNAs based on high-throughput sequencing studies; however, follow-up analyses by other groups have challenged the validity of many of these findings. A notable case is *N*^1^-methyladenosine (m^1^A) in mammalian mRNA. In 2016, two independent studies used antibody-based enrichment to map m^1^A across the transcriptome, identifying thousands of internal sites with moderate stoichiometries located throughout coding sequences and untranslated regions (Dominissini et al, 2016; Li et al, 2016). However, subsequent studies by us and others sought to validate these findings by measuring m^1^A-induced misincorporations in sequencing reads (Grozhik et al, 2019; Safra et al, 2017). These analyses revealed that m^1^A is almost entirely absent from cellular mRNA and that the antibody used in the original studies cross-reacted with the m^7^G cap structure, leading to artifactual enrichment at 5′ UTRs (Grozhik et al, 2019; Safra et al, 2017). These studies reported that m^1^A is primarily present at individual sites within the mitochondrial *ND5* mRNA, the lncRNA *MALAT1*, and *PRUNE1* mRNA, with a possibility of a small number of additional sites occurring at very low stoichiometries (Grozhik et al, 2019; Safra et al, 2017).

To resolve discrepancies in m^1^A detection, a recent study engineered a reverse transcriptase that selectively recognizes m^1^A by efficiently introducing signature misincorporations during cDNA synthesis. Specifically, this enzyme generates A-to-T transitions at m^1^A sites during sequencing, creating a distinct mutational signature (Zhou et al, 2019). Using this tool, the authors identified 45 high-confidence internal m^1^A sites in mammalian mRNAs. Aside from the previously validated *MALAT1*, *ND5* and *PRUNE1* sites, these newly reported sites were unexpected, as they were not detected in earlier studies that searched for m^1^A-induced misincorporations, albeit using standard reverse transcriptase that are less efficient at detecting m^1^A. Although this study appeared to definitively demonstrate that m^1^A is present at multiple sites throughout the transcriptome, this study, as with the previous m^1^A mapping studies, did not include biochemical validation of the specific identified m^1^A sites. This is largely because methods to site-specifically detect diverse modified nucleotides in mRNA are laborious. Thus, it remains unresolved whether the newly identified sites represent bona fide m^1^A modifications in vivo, or whether m^1^A is indeed primarily restricted to *MALAT1* lncRNA and the *ND5* and *PRUNE1* mRNAs.

Beyond m^1^A, *N*⁷-methylguanosine (m^7^G) has also been proposed to occur internally within mammalian mRNAs. m^7^G was mapped based on chemically induced m^7^G base conversions coupled with reverse transcriptase–dependent mutational signatures (Zhang et al, 2019). However, none of these sites have been independently validated using biochemical approaches. A separate group commented that the m^7^G signature mutations occurred preferentially at the ends of sequencing reads, a region known to have elevated error rates (Wiener and Schwartz, 2021). Another study employed miCLIP-seq-based approach and identified thousands of potential internal m^7^G clusters across the transcriptome (Malbec et al, 2019). However, the overlap between the sites reported in this study and those identified by Zhang et al was minimal, raising questions which dataset, if any, accurately identified m^7^G sites. Another group performed m^7^G mapping in yeast and *E. coli* mRNAs but was unable to detect any m^7^G modifications within mRNA at all (Enroth et al, 2019). Thus, questions have been raised whether internal m^7^G modifications truly exist in mRNA or whether the signals reflect methodological artifacts.

Many of the controversies surrounding low-abundance RNA modifications would not exist if studies routinely included site-specific biochemical validation to complement sequencing-based approaches. However, a major limitation in the field has been the lack of a non-laborious, generalizable method for such validation. To address this for m^6^A, we recently developed SCARPET (site-specific cleavage and radiolabeling followed by purification, exonuclease digestion, and thin-layer chromatography). SCARPET is a biochemical approach for detecting m^6^A at specific mRNA sites (Mirza et al, 2024). In the current study, we extend the SCARPET protocol to detect a broader panel of RNA modifications, including m^7^G, m^1^A, pseudouridine (Ψ), *N*⁶,*N*⁶-dimethyladenosine (m^6^_2_A), 5-methylcytosine (m^5^C), and inosine. The accuracy and sensitivity of SCARPET are substantially improved by selectively enriching for the mRNA containing the candidate modification. To enable this, we also established a simple and efficient RNA enrichment method using biotinylated oligonucleotides. Applying SCARPET, we provide biochemical evidence that m^7^G is indeed present as an internal modification at specific mRNA sites, with unexpectedly high stoichiometries. In contrast, we show that the recently reported m^1^A sites correspond instead to inosine. These sites were misclassified as m^1^A because A-to-G sequence changes induced by A-to-I RNA editing were included, rather than solely relying on the A-to-T mutations induced by the m^1^A-detecting reverse transcriptase. Together, these findings resolve outstanding questions regarding the presence of m^7^G and m^1^A in mRNA and establish SCARPET as a robust and versatile tool for site-specific validation of a wide range of nucleotide modifications.

## Results

### Optimization of SCARPET to detect diverse RNA modifications

We previously showed that SCARPET can detect and quantify the stoichiometry of specific m^6^A sites in the transcriptome (Mirza et al, 2024). In that study, SCARPET was optimized under TLC conditions specifically to resolve A from m^6^A. The procedure relies on RNase H-mediated cleavage at the 5′ side of the m^6^A residue, directed by a modified oligonucleotide that guides RNase H to the correct position in the RNA (Mirza et al, 2024).

**Figure 1:**
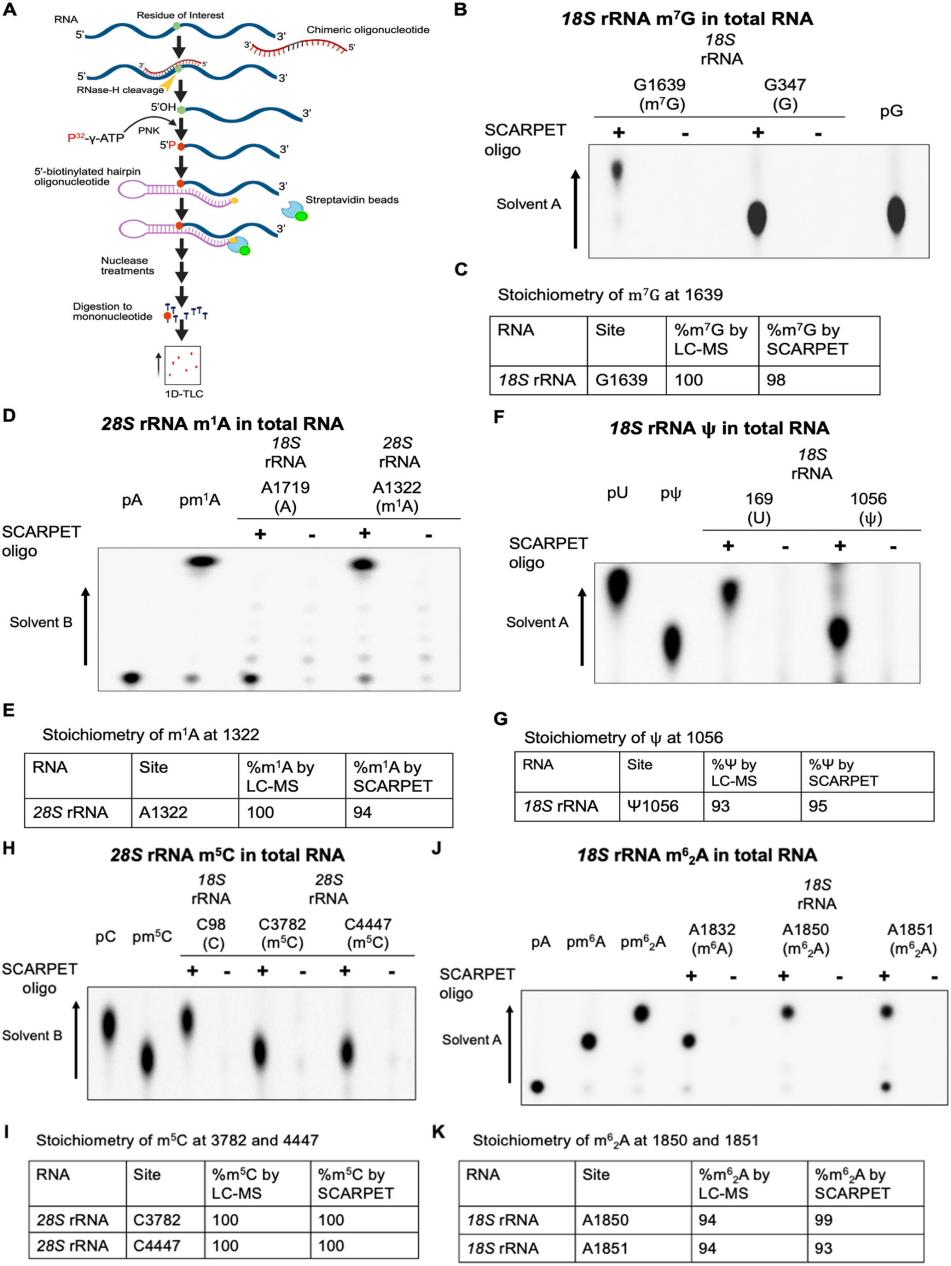
SCARPET detects diverse RNA modifications in rRNAs. (**A**) Schematic representation of the SCARPET assay workflow. (**B**) SCARPET detects internal m^7^G at position G1639 in *18S* rRNA, while unmodified G347 migrates with the guanosine (pG) standard (*N* = 3). (**C**) Table displaying stoichiometry of m^7^G at G1639 as determined by LC-MS (Taoka et al, 2018) and SCARPET. (**D**) SCARPET efficiently detects m^1^A at position 1322 in *28S* rRNA. Residue A1719 in *18S* rRNA, which contains unmodified adenosine, serves as a negative control (*N* = 3). (**E**) Table summarizing m^1^A stoichiometry at A1322 in *28S* rRNA as determined by LC-MS (Taoka et al, 2018) and SCARPET. (**F**) SCARPET detects Ψ at position 1056 in *18S* rRNA. A distinct spot from residue 1056 migrates in parallel with the Ψ standard, confirming the presence of pseudouridine. Unmodified uridine at position 169 serves as a negative control and migrates with the uridine standard (*N* = 3). (**G**) Table summarizing Ψ stoichiometry at position 1056 in *18S* rRNA as determined by LC-MS (Taoka et al, 2018) and SCARPET. (**H**) SCARPET detects m^5^C at two known sites, C3782 and C4447, in 28S rRNA. C98 from *18S* rRNA serves as a negative control and migrates in parallel with the unmodified cytidine standard (*N* = 2). (**I**) Table summarizing stoichiometries of m^5^C at C3782 and C4447 determined by LC-MS (Taoka et al, 2018) and SCARPET. (**J**) SCARPET detects m^6^_2_A at positions A1850 and A1851 in *18S* rRNA. SCARPET of A1832 shows presence of m^6^A at residue 1832 in *18S* rRNA (*N* = 3). (**K**) Table summarizing m^6^_2_A stoichiometry at A1850 and A1851 as measured by LC-MS (Taoka et al, 2018) and SCARPET. Source data are available online for this figure.

Although SCARPET is effective for detecting m^6^A, it remains unclear whether the method is compatible with other modified nucleotides. We therefore asked whether SCARPET can detect and quantify the stoichiometry of m^1^A, m^7^G, and additional modifications. We first tested SCARPET at the well-established m^7^G site 1639 in *18S* rRNA (Taoka et al, 2018). To perform SCARPET, a SCARPET oligonucleotide is needed in order to cleave the RNA at the 5’ side of G1639. As described previously (Mirza et al, 2024), the SCARPET oligonucleotide contains DNA nucleotides so that RNase H cleaves the RNA at the intended site. RNase H is guided to the RNA site by using four deoxyribonucleotides. The 5′-deoxyribonucleotide pairs with the nucleotide immediately upstream of the target site and directs RNase H to cleave at the 5′ side of the target residue. This cleavage places the modified nucleotide at the 5′ end of the RNA fragment, which is then radiolabeled using [γ-³²P] ATP and T4 polynucleotide kinase (Fig. 1A). In the SCARPET oligonucleotide, these deoxyribonucleotides are flanked by eight 2′-O-methylated ribonucleotides on each side, resulting in a 20-nucleotide chimeric sequence.

SCARPET uses a second step to selectively isolate the radiolabeled RNA fragment from the RNA pool. In this step, a biotinylated self-splinting oligonucleotide hybridizes to the cleaved target RNA and ligates to the radiolabeled 5′ end (Fig. 1A). The biotinylated oligonucleotide enables capture of the ligated product on streptavidin-coated beads for subsequent nuclease digestion and TLC. In SCARPET, the modified nucleotide is identified based on its mobility in TLC. Each modified nucleotide requires a standard that shows the position of the modified nucleotide relative to the unmodified nucleotide. To prepare the standard, we used a synthetic 10-nucleotide RNA oligonucleotide with the nucleotide of interest positioned at the 5′ end. In this way, the modified nucleotide can be radiolabeled by phosphorylation on the 5’ OH with [γ-³²P] ATP. The radiolabeled oligonucleotide is then digested to mononucleotides, which provides the modified nucleotide as a radiolabeled standard for TLC. Similar standards are prepared for the unmodified nucleotide.

We first confirmed that we could distinguish m^7^G from G. We used 1 µg of DNase-treated total RNA extracted from HEK293T cells and performed SCARPET analysis of G1639 in 18S. TLC was performed using standard SCARPET solvent conditions (“Solvent A,” isopropanol:concentrated HCl:water, 70:15:15, v/v/v) and produced a spot that migrated above the unmodified guanosine standard (Fig. 1B). The faster migration of m^7^G is consistent with its greater hydrophobicity relative to guanosine. Densitometric quantification of the m^7^G and G spots for G1639 indicated a modification stoichiometry of ~98%, in close agreement with LC-MS–based measurements of m^7^G (Taoka et al, 2018) at this position (Fig. 1C). A control SCARPET analysis was performed for G347 in 18S, an unmodified guanosine in *18S* rRNA. This control only showed a single spot consistent with unmodified G, based on its identical migration to the guanosine standard (Fig. 1B). These data support the specificity of SCARPET analysis for m^7^G. A synthetic m^7^G standard could not be included because *N*⁷-methylation is chemically labile and thus m^7^G-containing oligonucleotides undergo rapid depurination (Devereaux et al, 2019) under the conditions required for their synthesis. Therefore, G1639 in 18S rRNA, which is an established m^7^G site as confirmed by SCARPET and multiple other orthogonal approaches (D’Ambrosi et al, 2024; Zhang et al, 2022), was used as a positive reference in all m^7^G experiments.

**Figure EV1:**
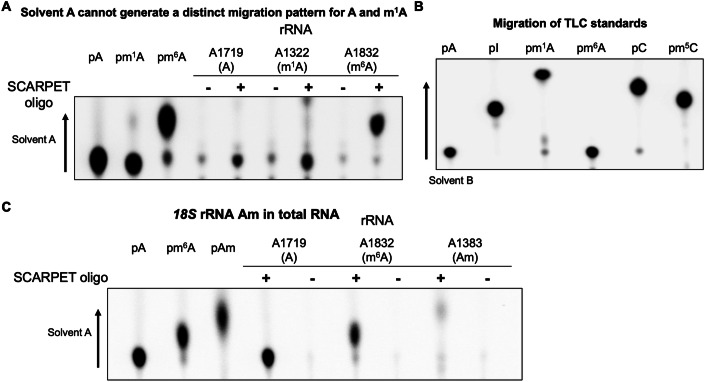
Solvent optimization improves resolution of RNA modifications by SCARPET. (**A**) SCARPET analysis using solvent A (Isopropanol: Concentrated HCl: Water, 70:15:15) does not generate distinct TLC migration profiles for adenosine (pA) and 1-methyladenosine (pm^1^A). (**B**) TLC separation of nucleotide standards using solvent B (saturated (ammonium sulfate:10 mM sodium acetate, pH 6.0: Isopropanol, 79:19:2) demonstrates improved resolution for Inosine (pI), pm^1^A, and pm^5^C, which migrate distinctly from their unmodified counterparts. (**C**) SCARPET analysis of residue A1383 in 18S rRNA shows a very faint signal at the Am position, indicating that SCARPET is largely ineffective at detecting 2′-O-methyl modifications.

We next asked if SCARPET can detect m^1^A. To test this, we performed SCARPET on A1322 in *28S* rRNA, a known m^1^A site which is modified at nearly 100% stoichiometry (Sharma et al, 2018; Taoka et al, 2018). However, when we tested the 5′-radiolabeled m^1^A and A standards, we found that they showed similar migration on TLC in the standard SCARPET solvent, making these nucleotides difficult to resolve (Fig. EV1A). To address this limitation, we screened solvent systems to improve separation. We identified an alternate solvent (“Solvent B”, saturated ammonium sulfate:10 mM sodium acetate, pH 6.0:isopropanol, 79:19:2, v/v/v), which clearly separated m^1^A from A (Figs. 1D and EV1B). Using this solvent, we performed SCARPET analysis of A1322 in *28S* rRNA, which showed that this site is m^1^A and it is modified at near-complete stoichiometry (Fig. 1E). These results demonstrate that SCARPET can reliably detect both m^1^A and m^7^G.

Although our focus was on m^1^A and m^7^G, we also examined whether SCARPET could detect additional RNA modifications. To test for Ψ, we performed SCARPET on U1056 in *18S* rRNA, a known Ψ site (Taoka et al, 2018). U1056 produced a spot that migrated identically to the Ψ standard (Fig. 1F). As a control, we analyzed U169, an unmodified uridine (U) in *18S* rRNA. The spot from U169 migrated at the same position as the U standard (Fig. 1F). Densitometric analysis estimated the modification stoichiometry of U1056 at ~95% (Fig. 1G), closely matching the previously reported LC-MS value of 93% (Taoka et al, 2018).

Next, we performed SCARPET on C3782 and C4447 in *28S* rRNA, which are known m^5^C sites (Taoka et al, 2018). In our experiments, Solvent B provided improved separation of C and m^5^C on TLC compared with the standard SCARPET solvent (Fig. EV1B). SCARPET analysis confirmed that both residues were fully methylated m^5^C sites, consistent with previous reports (Fig. 1H,I).

We next performed SCARPET on A1850 and A1851 in human *18S* rRNA, which are known to both be *N*⁶,*N*⁶-dimethyladenosine (m^6^_2_A) (Taoka et al, 2018). SCARPET analysis of A1850 and A1851 with Solvent A revealed TLC spots that migrated identically to the m^6^_2_A standard. Densitometric analysis showed that A1850 was modified to m^6^_2_A at ~99% stoichiometry, while at A1851, the m^6^_2_A stoichiometry was ~93% (Fig. 1J,K), consistent with previously reported LC-MS measurements (Taoka et al, 2018). Importantly, the m^6^_2_A spot was well resolved from both adenosine and the singly methylated m^6^A (Fig. 1J).

Lastly, we examined nucleotides carrying a 2′-*O*-methyl modification. Previous studies demonstrated that RNase H selectively recognizes the 2′-OH group, which ensures cleavage only at unmodified ribonucleotides (Nowotny et al, 2005). This implies that 2′-O-methylated nucleotides should resist cleavage. Nevertheless, to test whether SCARPET can detect such nucleotides, we performed SCARPET on A1383 in *18S* rRNA, which has been reported to be highly modified to Am (Taoka et al, 2018). SCARPET analysis detected a faint spot consistent with an unmodified A, and a very faint signal at the expected position of Am (Fig. EV1C). This suggests that the adenosine signal comes from the rare instances of unmodified A1383, while the faint Am signal likely represents the very low RNase H activity on Am. Overall, these results indicate that 2′-O-methylated nucleotides are largely refractory to SCARPET detection, consistent with their poor recognition by RNase H. Collectively, these results demonstrate that SCARPET can detect m^1^A, m^7^G, Ψ, m^6^_2_A, and m^5^C at single-nucleotide resolution and provide stoichiometric quantification.

### SCARPET accurately quantifies RNA modifications across a range of stoichiometries

Having established that SCARPET can detect and quantify multiple RNA modification types, we next asked whether the method is capable of accurately measuring modification stoichiometry at sites that are only partially modified. We previously demonstrated this for m^6^A using synthetic RNA titrations (Mirza et al, 2024); here, we extended that analysis to Ψ and m^1^A. To this end, we prepared synthetic RNA oligonucleotides bearing either a modified or unmodified residue at a specific site and mixed them at defined ratios spanning 0% to 100% modification in 20% increments.

**Figure 2:**
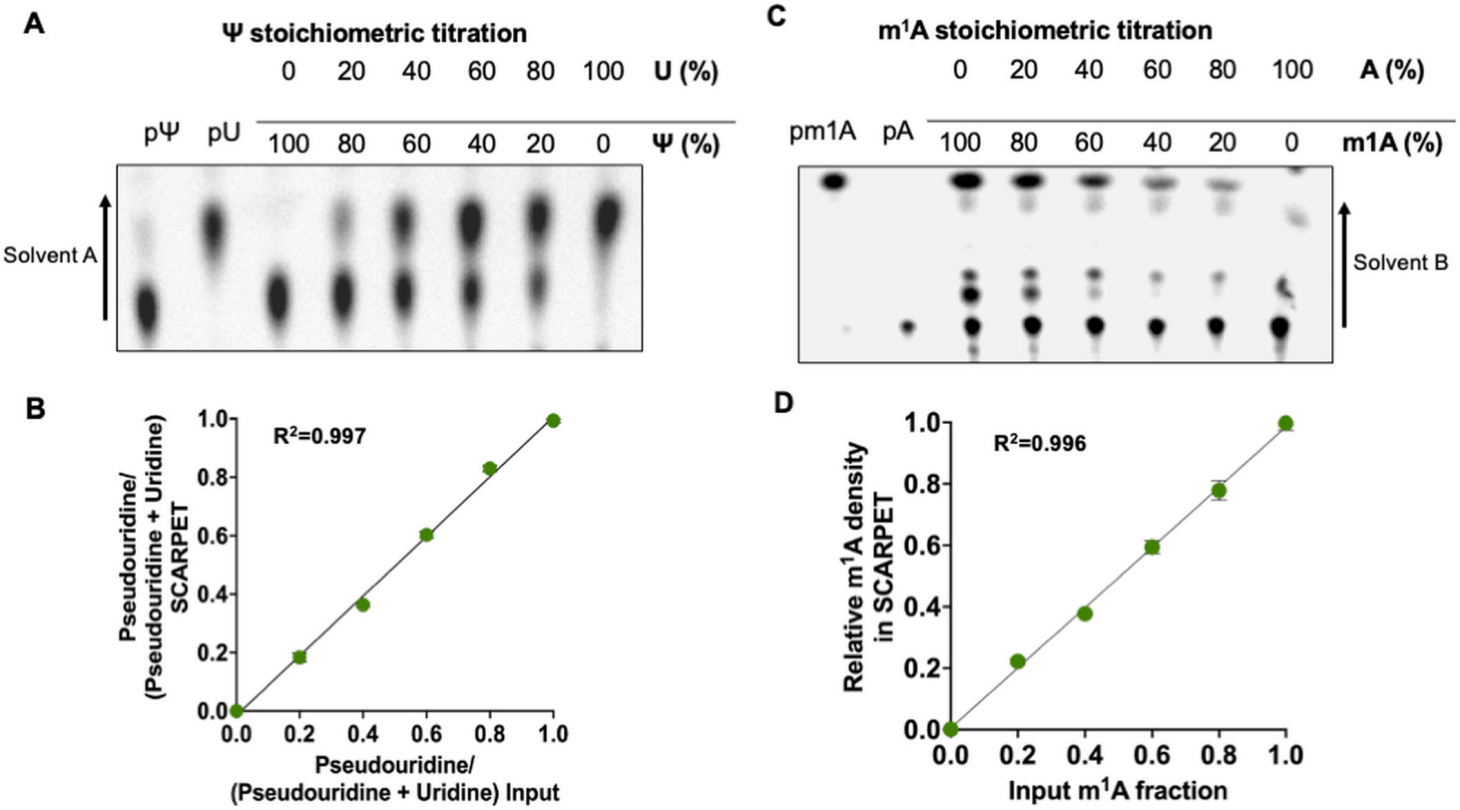
SCARPET accurately quantifies RNA modifications across a range of stoichiometries. (**A**) TLC analysis of SCARPET performed on synthetic RNA mixtures containing defined proportions of Ψ and U. The Ψ content of each lane ranges from 100% to 0% in 20% decrements (U content correspondingly ranges from 0% to 100%). pΨ and pU standards are shown on the left (*N* = 3). (**B**) Calibration curve comparing input Ψ fraction [Ψ/(Ψ + U)] (X axis) with the SCARPET-measured Ψ stoichiometry [Ψ/(Ψ + U)] (Y axis) across all mixture ratios tested. Each point represents the mean ± SD of three independent replicates. (**C**) TLC analysis of SCARPET performed on synthetic RNA mixtures containing defined proportions of m^1^A and unmodified adenosine. The m^1^A content of each lane ranges from 100% to 0% in 20% decrements. pm^1^A and pA standards are shown on the left (*N* = 3). (**D**) Calibration curve comparing input m^1^A fraction with relative m^1^A density as measured by SCARPET, normalized to the m¹A standard. Each point represents the mean ± SD of three independent replicates. Source data are available online for this figure.

We first tested Ψ by mixing Ψ- and U-containing synthetic RNAs at predefined ratios and performing SCARPET analysis. TLC analysis produced distinct Ψ and U spots at each mixture point, with the relative intensity of the Ψ spot progressively increasing as a function of the Ψ input fraction (Fig. 2A). Densitometric quantification of both spots showed that the SCARPET-measured Ψ stoichiometry [Ψ/(Ψ + U)] closely matched the known input fraction across the entire range tested, yielding a near-perfect linear relationship (Fig. 2B). These results demonstrate that SCARPET provides accurate stoichiometric measurements for Ψ at levels as low as 20%. We performed an analogous titration experiment for m^1^A by mixing a m^1^A-containing synthetic RNA with the corresponding unmodified adenosine RNA. TLC analysis revealed the expected pm^1^A spot at all mixture points (Fig. 2C); however, the intensity of the adenosine spot does not scale proportionally with the input A fraction. It is possible that a fraction of m^1^A spontaneously undergoes Dimroth rearrangement generating m^6^A, which then co-migrates with adenosine under Solvent B conditions (Fig. EV1B), artificially inflating the adenosine signal. Nevertheless, we quantified m^1^A detection performance by measuring the density of the m^1^A spot alone, normalized to the signal in the 100% m^1^A sample. The relative m^1^A density increased linearly with input m^1^A fraction across the full range tested (Fig. 2D), confirming that SCARPET signal faithfully detects m^1^A levels even at low stoichiometries, although calculating absolute m^1^A stoichiometries might not be possible. Taken together, these data, along with the previously demonstrated ability of SCARPET to quantitatively assess m^6^A stoichiometry (Mirza et al, 2024), establish that SCARPET can detect and accurately quantify diverse RNA modifications across a broad stoichiometric range.

### Targeted RNA capture using BioCapT

We previously showed that the quantitative accuracy of SCARPET for measuring m^6^A at specific sites improves when performed with purified target mRNA instead of poly(A)-enriched RNA. Poly(A)-enriched RNA was associated with a higher adenosine background, leading to underestimation of m^6^A stoichiometry (Mirza et al, 2024). This background arises from off-target hybridization and RNase H-mediated cleavage by the SCARPET oligonucleotide, which increases 5′ adenosine signals from across the transcriptome. Purifying the target mRNA reduces the opportunity for off-target cleavage, thus lowering the background adenosine and improving the accuracy of m^6^A stoichiometry measurements (Mirza et al, 2024).

**Figure 3:**
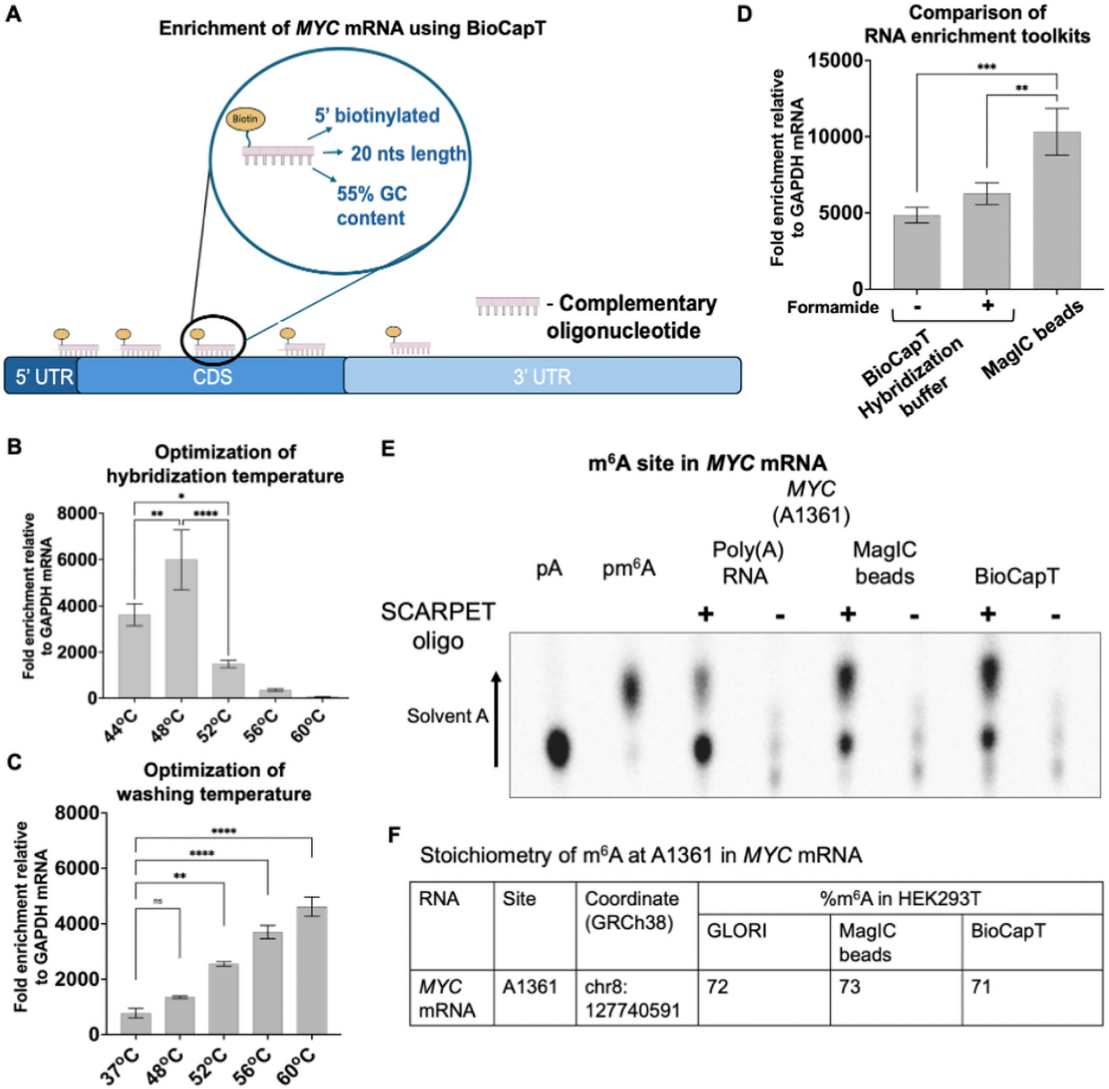
Design and optimization of BioCapT RNA enrichment using biotinylated DNA probes. (**A**) Schematic representation of *MYC* mRNA enrichment with BioCapT strategy. Oligonucleotides were 20 nucleotides in length, had 55% GC content, and were biotinylated at the 5′ end. Probes were designed to target multiple regions across the *MYC* transcript for efficient capture. (**B**) Optimization of hybridization temperature for the enrichment of endogenous *MYC* mRNA using BioCapT. Fold enrichment was quantified by RT-qPCR relative to *GAPDH* mRNA. Capture efficiency peaked at 48 °C, with significant reduction at both lower and higher temperatures. Error bars indicate standard deviation (SD). Data represent mean ± SD from three independent biological replicates (*N* = 3). Statistical significance was determined using ordinary one-way ANOVA followed by Tukey’s multiple comparisons test. Adjusted *P* values: 44 °C vs 48 °C, *P* = 0.0058; 44 °C vs 52 °C, *P* = 0.0122; 44 °C vs 56 °C, *P* = 0.0005; 44 °C vs 60 °C, *P* = 0.0003; 48 °C vs 52 °C, *P* < 0.0001; 48 °C vs 56 °C, *P* < 0.0001; 48 °C vs 60 °C, *P* < 0.0001; 52 °C vs 56 °C, *P* = 0.2335 (ns); 52 °C vs 60 °C, *P* = 0.0989 (ns); 56 °C vs 60 °C, *P* = 0.9742 (ns). Significance levels: *P < 0.05, **P < 0.01, ***P < 0.001, ****P < 0.0001; ns = not significant. (**C**) Optimization of washing temperature. Increasing washing temperatures improved enrichment specificity by reducing non-specific RNA carryover, with highest enrichment observed at 60 °C. Error bars indicate SD. Data represent mean ± SD from three independent biological replicates (*N* = 3). Statistical significance was determined using ordinary one-way ANOVA followed by Dunnett’s multiple comparisons test against the 37 °C condition. Adjusted *P* values: 37 °C vs 48 °C, *P* = 0.8670 (ns); 37 °C vs 52 °C, *p* = 0.0031; 37 °C vs 56 °C, *P* < 0.0001; 37 °C vs 60 °C, *P* < 0.0001. Significance levels: *P < 0.05, **P < 0.01, ***P < 0.001, ****P < 0.0001; ns = not significant. (**D**) Comparison of BioCapT enrichment performance in different buffers and with Magic Beads RNA capture kit for enrichment of endogenous *MYC* mRNA. Error bars indicate SD. Data represent mean ± SD from three independent biological replicates (*N* = 3). Statistical significance was determined using ordinary one-way ANOVA followed by Tukey’s multiple comparisons test. Adjusted *P* values: Hybridization buffer vs Formamide hybridization buffer, *P* = 0.0744 (ns); Hybridization buffer vs ElementZero MaGIC beads, *P* = 0.0002; Formamide hybridization buffer vs ElementZero MaGIC beads, *P *= 0.0019. Significance levels: *P < 0.05, **P < 0.01, ***P < 0.001, ****P < 0.0001; ns = not significant. (**E**) SCARPET analysis of the m^6^A site at position A1361 in *MYC* mRNA. The assay was performed using different RNA inputs: poly(A)-purified mRNA, *MYC* mRNA enriched using either MagIC beads or custom protocol described in this study (*N* = 2). (**F**) Table summarizing the m^6^A stoichiometry at A1361 in *MYC* mRNA, as measured by GLORI (Liu et al, 2023), and SCARPET with different RNA enrichment methods. Source data are available online for this figure.

**Figure EV2:**
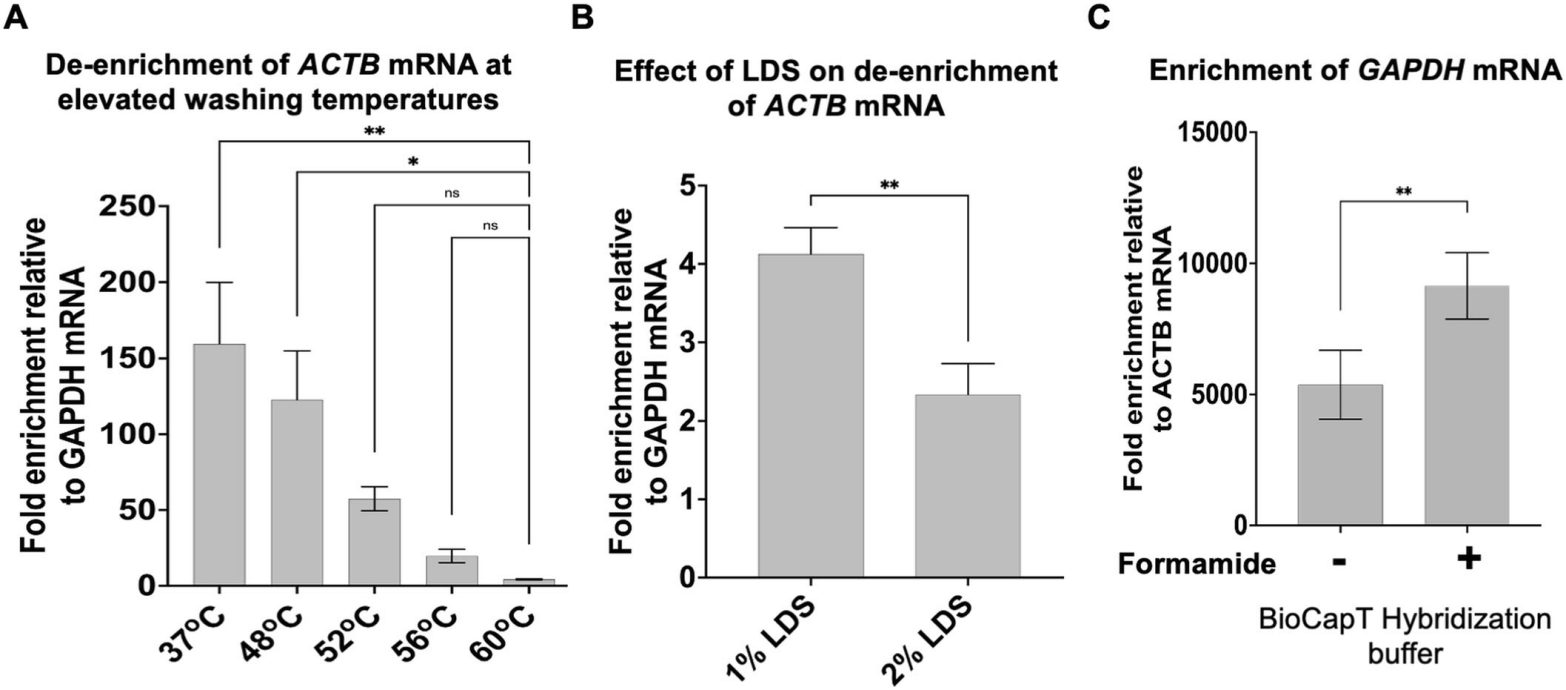
Optimization of washing stringency enhances transcript-specific RNA enrichment by reducing non-specific background. (**A**) Relative levels of non-target *ACTB* mRNA compared with *GAPDH* mRNA decreased with elevated washing temperatures in BioCapT protocol for *MYC* mRNA enrichment. Error bars indicate SD. Data represent mean ± SD from three independent biological replicates (*N* = 3). Statistical significance was determined using ordinary one-way ANOVA followed by Tukey’s multiple comparisons test. Adjusted *P* values: 60 °C vs 37 °C, *P* < 0.0001; 60 °C vs 48 °C, *P* = 0.0003; 60 °C vs 52 °C, *P* = 0.0096; 60 °C vs 56 °C, *P *= 0.8382 (ns). Significance levels: *P < 0.05, **P < 0.01, ***P < 0.001, ****P < 0.0001; ns = not significant. (**B**) Increasing the LDS concentration from 1% to 2% during wash steps significantly reduced *ACTB* levels (**P < 0.01, unpaired *t* test) in BioCapT enriched *MYC* mRNA. Error bars indicate SD. Data represent mean ± SD from three independent biological replicates (*N* = 3). (**C**) Enrichment of *GAPDH* mRNA relative to *ACTB* mRNA using BioCapT protocol for *GAPDH* mRNA enrichment (**P < 0.01, unpaired *t* test). Error bars indicate SD. Data represent mean ± SD from three independent biological replicates (*N* = 3).

We previously used a commercial RNA purification kit customized for the *MYC* mRNA to obtain mRNA for SCARPET (Mirza et al, 2024). Since this can be cost-prohibitive when analyzing multiple RNAs, we sought to develop a rapid, cost-effective enrichment strategy that yields sufficient mRNA for SCARPET. Several studies have used complementary oligonucleotides for transcript-specific enrichment, but reported efficiencies have varied widely, ranging from ~200-fold to >1000-fold depending on probe design, transcript abundance, and protocol stringency (Chu et al, 2021; Engreitz et al, 2014; Pant and Kumarswamy, 2024; Spiniello et al, 2019). To address this limitation, we optimized BioCapT (Biotinylated oligonucleotide Capture of Transcripts) RNA enrichment strategy for *MYC* mRNA, a relatively low-abundance transcript (Dani et al, 1984). We designed seven 20-nucleotide DNA oligonucleotides with a G/C content of 55% to target regions distributed across the *MYC* transcript (Fig. 3A). The complementary oligonucleotides were 5’-biotinylated to facilitate the downstream pulldown. We used a buffer containing Li⁺ ions, which stabilize nucleic acid duplexes and minimizes the formation of secondary structures such as G-quadruplexes (Arteaga et al, 2024). We systematically optimized hybridization and washing conditions to maximize capture specificity and yield. Hybridization at 48 °C for 1 h produced the highest capture of *MYC* mRNA (Fig. 3B). Washing at elevated temperatures and with high concentrations of lithium dodecyl sulfate (LDS) enhanced removal of non-specific RNAs without compromising target (*MYC* mRNA) enrichment (Fig. 3C). We also observed marked depletion of non-target *ACTB* (*β-actin*) mRNA at higher washing temperatures (Fig. EV2A), which further increased at higher LDS concentrations (Fig. EV2B). Addition of formamide further increased hybridization stringency and markedly improved capture efficiency for *MYC* mRNA (Fig. 3D). The optimized BioCapT protocol is described in detail in “Methods”.

We compared our BioCapT protocol with a commercial kit for purification of *MYC* mRNA (ElementZero MagIC beads kit), which we had used previously (Mirza et al, 2024). The MagIC beads kit achieved ~10,000-fold enrichment of *MYC* relative to *GAPDH* mRNA, while our protocol achieved ~6,000-fold enrichment (Fig. 3D). We also applied our BioCapT to enrich *GAPDH* mRNA, a relatively abundant transcript. In this case, BioCapT yielded ~10,000-fold enrichment relative to *ACTB* mRNA (Fig. EV2C). To determine whether this enrichment was sufficient for SCARPET, we performed SCARPET on A1361 in *MYC* mRNA, which is a high stoichiometry m^6^A site (Mirza et al, 2024). When poly(A) RNA was used, the major signal was from the adenosine spot, with a faint m^6^A signal (Fig. 3E). However, when BioCapT-enriched transcript was used, the m^6^A spot was much more prominent (Fig. 3E), and the calculated m^6^A stoichiometry closely matched the m^6^A stoichiometry using *MYC* mRNA purified with the MagIC beads, and with the previously reported m^6^A stoichiometry for this site (Liu et al, 2023) (Fig. 3F). Together, these results indicate that BioCapT can be used for rapid and simple transcript enrichment to increase the accuracy of SCARPET-based stoichiometric quantification.

### Internal m^7^G occur in mammalian mRNAs with varying stoichiometries

**Figure 4:**
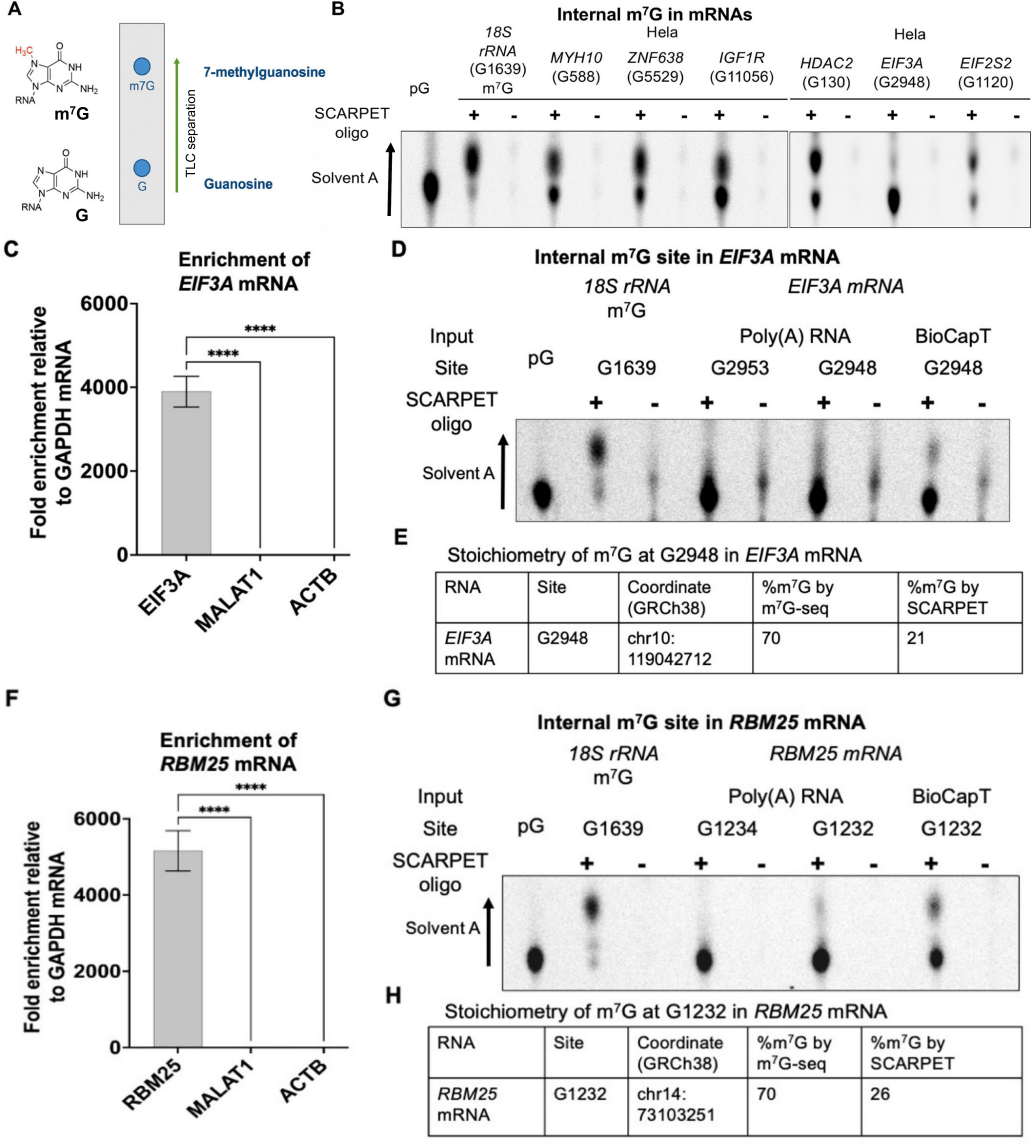
SCARPET detects internal m^7^G residues in mammalian mRNAs. (**A**) Schematic illustrating the chemical structures of guanosine (G) and N7-methylguanosine (m^7^G), and their separation by TLC in SCARPET. (**B**) SCARPET analysis of six putative internal m^7^G sites in human mRNAs revealed that most candidates produced a TLC spot that co-migrated with the known m^7^G reference from *18S* rRNA (G1639) (*N* = 3). (**C**) RT-qPCR analysis showing specific enrichment of *EIF3A* mRNA relative to *GAPDH* mRNA using the custom RNA capture protocol. *MALAT1* and *ACTB* mRNAs serve as non-targeted controls. Error bars indicate SD. Data represent mean ± SD from three independent biological replicates (*N* = 3). Statistical significance was determined using ordinary one-way ANOVA followed by Dunnett’s multiple comparisons test against *EIF3A*. Adjusted *P* values: *EIF3A* vs *MALAT1*, *P* < 0.0001; *EIF3A* vs *ACTB*, *P* < 0.0001. (**D**) SCARPET analysis of the putative m^7^G site at G2948 in *EIF3A* mRNA using either poly(A)-purified mRNA or enriched *EIF3A* mRNA as input for SCARPET. G2953, an adjacent unmodified guanosine within *EIF3A* mRNA, serves as a negative control and produces only a G spot with no detectable m^7^G signal (*N* = 3). (**E**) Table comparing the estimated m^7^G stoichiometry at G2948 by m^7^G-seq (Zhang et al, 2019) and SCARPET. (**F**) RT-qPCR analysis showing specific enrichment of *RBM25* mRNA relative to *GAPDH* mRNA using the custom RNA capture protocol. *MALAT1* and *ACTB* mRNAs serve as non-targeted controls. Error bars indicate SD. Data represent mean ± SD from three independent biological replicates (*N* = 3). Statistical significance was determined using ordinary one-way ANOVA followed by Dunnett’s multiple comparisons test against *RBM25*. Adjusted *P* values: *RBM25* vs *MALAT1*, *P* < 0.0001; *RBM25* vs *ACTB*, *P *< 0.0001. (**G**) SCARPET analysis of the putative m^7^G site at G1232 in *RBM25* mRNA using either poly(A)-purified mRNA or enriched *RBM25* mRNA as input for SCARPET. An adjacent unmodified guanosine, G1234, within *RBM25* mRNA, serves as a negative control and produces only a single spot migrating parallel to G standard, with no detectable m^7^G signal (*N* = 3). (**H**) Table comparing the estimated m^7^G stoichiometry at G1232 by m^7^G-seq (Zhang et al, 2019) and SCARPET. Source data are available online for this figure.

**Figure EV3:**
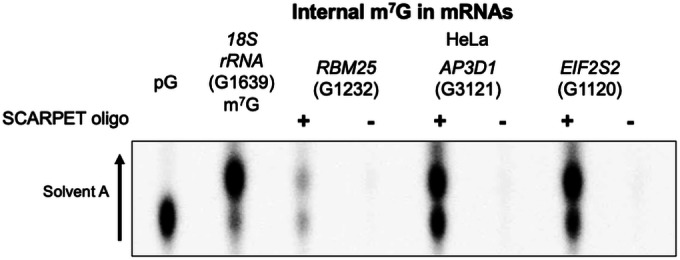
SCARPET detects internal m^7^G in *RBM25*, *AP3D1*, and *EIF2S2* mRNAs. SCARPET validation of candidate internal m^7^G sites in *RBM25* (G1232), *AP3D1* (G3121), and *EIF2S2* (G1120) mRNAs in HeLa cells. G1639 in *18S* rRNA served as a positive control for internal m^7^G (*N* = 2).

We next applied SCARPET to test the putative m^7^G sites reported by Zhang et al (Zhang et al, 2019). In this study, the internal m^7^G sites were based on the sites that were found in common using two orthogonal techniques, m^7^G-MeRIP-seq and m^7^G-seq, both applied to HeLa and HepG2 cells mRNAs (Zhang et al, 2019). We selected eight high confidence sites identified by Zhang et al and designed SCARPET oligonucleotides for each. For an initial assessment, we performed SCARPET using poly(A)-enriched RNA from HeLa cells, without prior mRNA purification, to test for the presence of m^7^G. For each site, SCARPET revealed a spot corresponding to G and a second distinct TLC m^7^G spot that co-migrated with the m^7^G standard (Fig. 4A**)**. Thus, SCARPET confirmed that the eight tested sites represent bona fide internal m^7^G modification sites (Figs. 4B and EV3).

We next selected some m^7^G sites for SCARPET analysis using the purified mRNA for more accurate stoichiometry measurements. We focused on *EIF3A* and *RBM25* mRNAs, which both showed comparatively weak signals for m^7^G (Figs. 4B and EV3). Both sites were reported as high-confidence, high-stoichiometry m^7^G sites (Zhang et al, 2019). Using BioCapT, we achieved ~4000-fold enrichment for *EIF3A* mRNA (Fig. 4C) and ~5000-fold for *RBM25* mRNA (Fig. 4F), relative to *GAPDH* mRNA. Following enrichment, the m^7^G spots became markedly stronger (Fig. 4D,G), with measured stoichiometries of ~21% for *EIF3A* (Fig. 4E) and ~26% for *RBM25* (Fig. 4H). To rule out the possibility that the observed m^7^G signals represent a systematic artifact, rather than bona fide internal modifications, we included adjacent unmodified guanosine residues within the same transcripts as negative controls. Specifically, we performed SCARPET on G2953 in *EIF3A* mRNA and G1234 in *RBM25* mRNA, sites immediately neighboring the putative m^7^G positions. In both cases, SCARPET produced only a single spot co-migrating with the unmodified G standard, with no detectable m^7^G signal (Fig. 4D,G). This site-specificity confirms that the m^7^G signals at G2948 in *EIF3A* mRNA and G1232 in *RBM25* mRNA reflect genuine internal modifications rather than a non-specific or cap-derived artifact. Overall, these results validate internal m^7^G sites in mammalian mRNAs and support the view that internal m^7^G occurs in mRNAs at appreciable stoichiometries.

### SCARPET reveals that high stoichiometry m^1^A is rare in mammalian mRNAs

We next tested internal m^1^A sites in mammalian mRNAs, which remain a subject of active debate. The debate centers on whether m^1^A sites will always introduce misincorporations during cDNA synthesis. Two groups reported a lack of misincorporations in cellular RNA, including RNA immunoprecipitated with m^1^A antibodies (Grozhik et al, 2019; Wiener and Schwartz, 2021). If m^1^A does not always introduce misincorporations, then the conclusion that m^1^A is primarily limited to *MALAT1*, *ND5*, and *PRUNE1* RNAs could be a substantial underestimation of m^1^A in the transcriptome. To address this, Zhou et al evolved a reverse transcriptase that causes highly efficient A-to-T misincorporations upon encountering an m^1^A residue (Zhou et al, 2019). Using this method, they reported 45 high confidence m^1^A sites, along with known sites in the *MALAT1* ncRNA and the *ND5*, and *PRUNE1* mRNAs.

**Figure 5:**
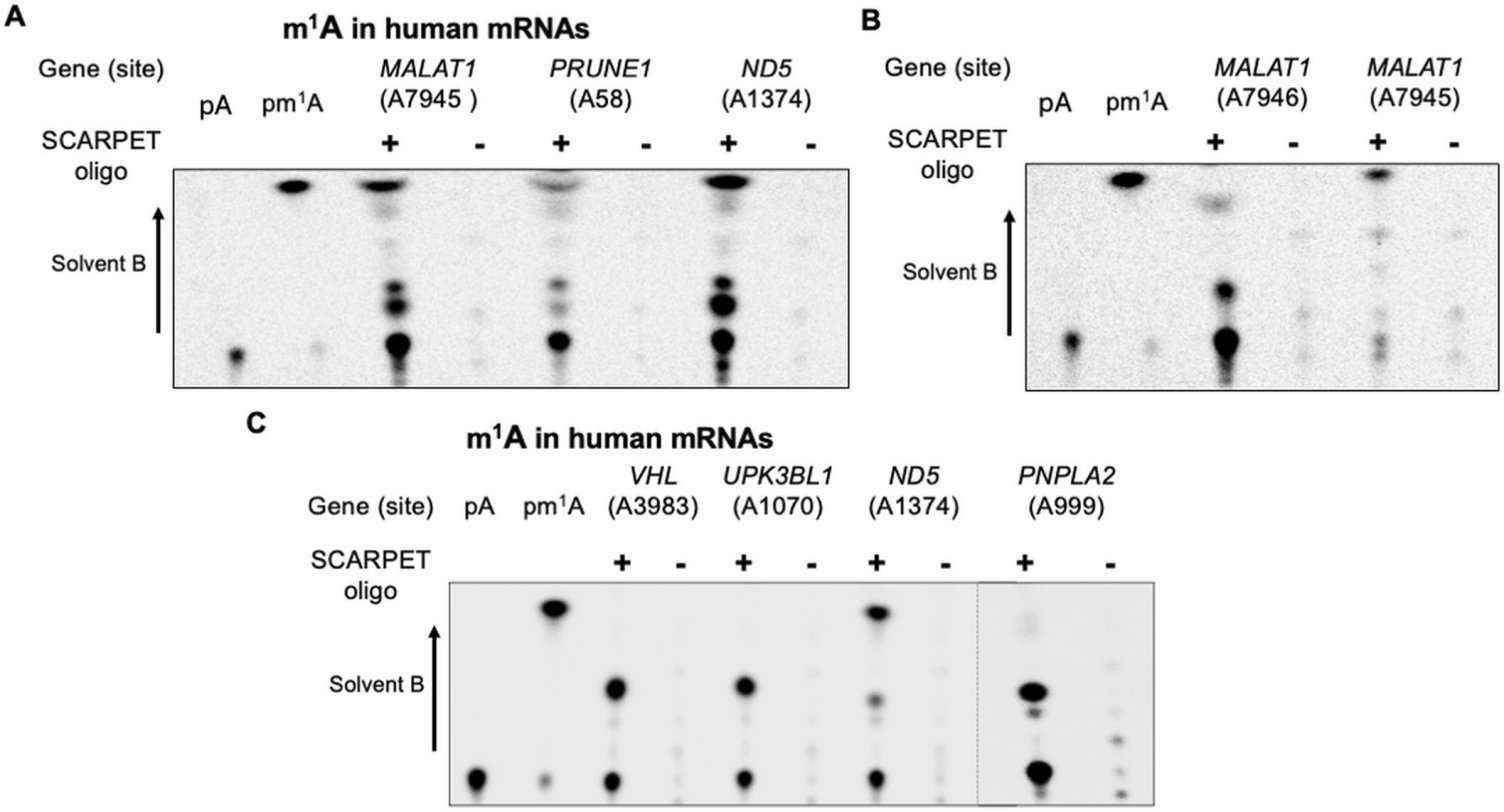
SCARPET reveals that internal m^1^A is rare in mammalian mRNAs. (**A**) SCARPET analysis of the known m^1^A sites in *MALAT1* (A7945), *PRUNE1* (A58), and *ND5* (A1374) RNAs revealed that these RNAs indeed harbor bona fide m^1^A sites (*N* = 2). (**B**) SCARPET analysis of an unmodified adenosine in *MALAT1* (A7946) did not produce m^1^A signal (*N* = 2). (**C**) SCARPET analysis of the putative m^1^A sites in *VHL* (A3983), *UPK3BL1* (A1070), and *PNPLA2* mRNAs did not produce a signal corresponding to m^1^A (*N* = 3). Source data are available online for this figure.

We therefore sought to biochemically validate both known and newly reported m^1^A sites in mammalian RNA. We first checked if SCARPET could detect m^1^A at three well-established sites: A7945 in *MALAT1* lncRNA, A1374 in *ND5* mRNA, and the A58 in *PRUNE1* mRNA, all of which are known to harbor m^1^A at high stoichiometry. SCARPET analysis of each site produced a spot migrating parallel to the m^1^A standard confirming the presence of m^1^A at these sites (Fig. 5A). An unmodified adenosine site in *MALAT1* (A7946) produces a spot co-migrating with the unmodified A standard, with no m^1^A signal, confirming the specificity of SCARPET for detecting m^1^A (Fig. 5B). We next examined some of the newly reported m^1^A sites, selecting *VHL*, *UPK3BL1*, and *PNPLA2* for biochemical validation, as these were predicted to harbor m^1^A at high stoichiometry (Zhou et al, 2019), and including *ND5* as a positive control. SCARPET analysis of the m^1^A site in *ND5* mRNA showed two clear spots, one corresponding to unmodified adenosine and the other migrating parallel to the m^1^A standard (Fig. 5C), confirming the presence of m^1^A in *ND5* mRNA. However, SCARPET analysis of the putative m^1^A sites in *VHL*, *UPK3BL1*, and *PNPLA2* produced a spot that matched adenosine, and surprisingly, a second spot that did not co-migrate with the m^1^A standard. No spot was detected at the position corresponding to m^1^A, indicating that m^1^A is absent at these sites (Fig. 5C). Taken together, these data suggest that a modification is present at these sites, but it is not m^1^A.

### A-to-I RNA editing accounts for misannotated m^1^A sites in human mRNAs

**Figure 6:**
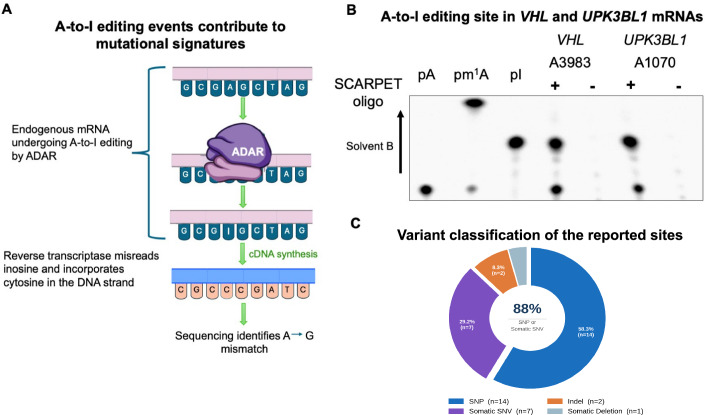
A-to-I editing underlies misidentification of m^1^A sites in mRNAs. (**A**) Schematic illustrating how A-to-I RNA editing generates mutational signatures in sequencing-based approaches. ADAR-mediated editing converts adenosine to inosine in endogenous mRNA. During cDNA synthesis, reverse transcriptase misreads inosine as guanosine, incorporating cytosine in the cDNA strand. This produces an apparent A-to-G transition in sequencing reads. (**B**) SCARPET analysis of A-to-I editing sites at A3983 in *VHL* mRNA and A1070 in *UPK3BL1* mRNA. TLC spots from both sites co-migrate with the pI (inosine) standard under Solvent B conditions, confirming the presence of inosine at these sites (*N* = 3). (**C**) Classification of genetic variants at the remaining 24 putative m¹A sites reported by Zhou et al (2019). The majority of these sites harbor known genomic variants. Source data are available online for this figure.

SCARPET analysis of *VHL*, *UPK3BL1*, and *PNPLA2* revealed the presence of a mystery spot at each site that did not align with adenosine or m^1^A standards (Fig. 5C). We next sought to identify the modification present at these positions. Several methyl modifications of adenosine in mRNAs are known, including m^6^A, *N*^6^, 2’-*O*-methyladenosine (m^6^A_m_), 2’-*O*-methyladenosine (A_m_), and inosine (I). However, SCARPET is unable to detect 2’-*O*-methyladenosine modifications, while m^6^A and adenosine shows similar migration in Solvent B (Fig. EV1B,C). We therefore considered inosine, which is formed as a result of A-to-I editing, and is known to generate a mutational signature during reverse transcription (Fig. 6A). Strikingly, the mystery spot co-migrated with the inosine standard (Fig. 6B), suggesting that these residues are inosine rather than m^1^A.

Inosine should be readily distinguished from m^1^A since inosine produces a characteristic A-to-G sequence change during sequencing, rather than the A-to-T mutation that is induced by the evolved reverse transcriptase. Reanalysis of the dataset reported by Zhou et al (Wiener and Schwartz, 2021) indicated that out of 45 new sites, 34 exhibit predominantly A-to-G mutations, nine are A-to-C mutations and only two are A-to-T mutations. Thus, the mutations used to identify *VHL*, *UPK3BL1*, and *PNPLA2* are consistent with inosine. To further support that these sites are inosine rather than m^1^A, we queried two A-to-I editing databases: REDIportal (D’Addabbo et al, 2024) and DARNED (Kiran et al, 2013). Both A3983 in *VHL* and A1070 in *UPK3BL1* were annotated as inosine in REDIportal. Among the other m^1^A sites identified by Zhou et al, 18 were identified as inosine in REDIportal. To assess the broader prevalence of this misannotation, we examined the variant landscape at the remaining 24 sites. This analysis revealed that the majority of these sites harbor SNPs or somatic SNVs (Fig. 6C; Dataset EV1), consistent with genomic variants rather than epitranscriptomic modifications. Together, these results indicate that internal m¹A in mammalian mRNAs has been substantially overestimated due to the confounding effect of A-to-I editing on sequencing-based modification maps.

## Discussion

Several nucleotide modifications have been proposed to occur widely in mammalian mRNAs. In many cases, however, the evidence has been based primarily on sequencing signals that lack biochemical validation. The resulting uncertainty has been most intense for m^1^A. Initial transcriptome-wide m^1^A maps relied on antibody-based enrichment of m^1^A-containing fragments and reported thousands of sites across coding sequences and untranslated regions (Dominissini et al, 2016; Li et al, 2016). Later studies challenged these findings by examining RNA sequences immunoprecipitated with m^1^A antibodies for characteristic m^1^A-induced misincorporations and found that these were largely absent (Safra et al, 2017). This raised questions about whether the antibody was indeed binding m^1^A or some other feature. We showed that the enrichment of m^1^A signals near the 5′ ends of transcripts resulted from cross-reactivity of the m^1^A antibody with the m^7^G cap rather than the presence of internal m^1^A (Grozhik et al, 2019). Nonetheless, the possibility remained that m^1^A could occur internally within mRNA at sites difficult for standard reverse transcriptase to detect. Our data indicate that all or most of the putative m^1^A sites are not m^1^A but mostly reflect inosines that were misannotated as m^1^A. Overall, our data suggest that m^1^A is not a widespread modification but instead is limited to just the *PRUNE1* mRNA, *ND5* mRNA and *MALAT1* lncRNA in mammalian cells.

A major advance for mapping m^1^A was the development of a reverse transcriptase that introduces highly efficient A-to-T transitions when encountering m^1^A (Zhou et al, 2019). This enzyme should reveal m^1^A in a broad range of sequence contexts. Zhou et al reported dozens of previously unrecognized internal m^1^A sites (Zhou et al, 2019). We used SCARPET to test these specific sites biochemically at the single-nucleotide level. SCARPET unambiguously identified m^1^A in *ND5* and *PRUNE1* mRNA, and in *MALAT1* lncRNA, in agreement with previous studies (Grozhik et al, 2019; Safra et al, 2017). In contrast, the sites reported in *VHL*, *UPK3BL1*, and *PNPLA2* did not contain detectable m^1^A. Instead, for *VHL* and *UPK3BL1* sites, SCARPET showed co-migration with inosine standards. Database searches further revealed that a majority of the newly reported m^1^A sites correspond to known or predicted A-to-I editing events. These results strongly support the conclusion that m^1^A is not widespread in mammalian mRNAs but is instead largely restricted to the previously validated sites in *PRUNE1*, *ND5* and *MALAT1* RNA.

The evolved reverse transcriptase developed by Zhou et al successfully detected the previously validated m^1^A sites, including sites in *PRUNE1*, *ND5*, and *MALAT1* RNA, indicating that the engineered enzyme is capable of m^1^A detection. However, the same approach also identified additional sites that, based on our biochemical analyses, do not contain m^1^A. Because the evolved reverse transcriptase was specifically engineered to induce A-to-T transversions in response to m^1^A (Zhou et al, 2019), it remains unclear why A-to-G transitions, which is associated with inosine (Gabay et al, 2022), were annotated as m^1^A. Nevertheless, it is clear that the evolved reverse transcriptase itself is highly effective for detecting bona fide m^1^A sites when using A-to-T transversions. The inability of the evolved reverse transcriptase to detect A-to-T transversions in highly sequenced cellular mRNA, other than in *PRUNE1*, *ND5*, and MALAT1, should be interpreted to mean that m^1^A is essentially nonexistent in cellular mRNA. The biochemical validation presented here, along with subsequent identification of these sites in databases of known RNA editing sites, suggests that most of the new m^1^A sites are instead inosine. Our biochemical conclusion is consistent with, and provides direct experimental support for the prior computational reanalysis by Wiener and Schwartz (Wiener and Schwartz, 2021), who independently reached a similar conclusion based on the predominance of A-to-G rather than A-to-T mutational signatures at these sites. However, cross-referencing with REDIportal and DARNED show that only 20 of the misannotated m^1^A sites are annotated A-to-I editing events. For the remaining sites that lacked an editing annotation, we found that most harbor known SNPs or somatic variants in dbSNP and COSMIC, indicating that the observed sequence heterogeneity reflects genomic variation rather than epitranscriptomic modification. These findings underscore a broader concern: sequencing-based modification mapping methods can be confounded by any source of sequence variation, including RNA editing and germline or somatic polymorphisms, and biochemical validation is therefore essential to distinguish true modifications from such confounders. Overall, other than tRNA and rRNA, m^1^A appears to be limited to three RNAs.

Internal m^7^G has also been unclear. Two independent sequencing approaches identified high confidence m^7^G sites, but other studies did not detect internal m^7^G, while another raised concerns about technical artifacts (Enroth et al, 2019; Wiener and Schwartz, 2021). SCARPET now provides biochemical confirmation that internal m^7^G occurs within specific mRNAs and can reach surprisingly high stoichiometries. The specificity of the m⁷G signal was further demonstrated by SCARPET analysis of adjacent unmodified guanosine residues within the same transcripts, where each produced only a single spot co-migrating with the unmodified G standard, with no detectable m⁷G. This site-specificity rules out the possibility that the m⁷G signals arise from cap-derived contamination or non-specific artifacts, a concern that had been raised by previous studies (Wiener and Schwartz, 2021). m^7^G therefore represents a genuine internal modification in mRNA. Notably, the m⁷G stoichiometries measured by SCARPET for *EIF3A* (~ 21%) and *RBM25* (~ 26%) are lower than those reported by Zhang et al (Zhang et al, 2019) using sequencing-based approaches. This quantitative difference likely reflects fundamental differences between the two methods. Zhang et al inferred stoichiometry from the frequency of chemically induced mutational signatures, an indirect readout that depends on reverse-transcription fidelity, local sequence context, and the computational model used to convert mutation rates into modification fractions. In contrast, SCARPET directly resolves modified and unmodified nucleotides by thin-layer chromatography and quantifies their relative abundance by densitometry. SCARPET-based stoichiometries for rRNA m^7^G site show close agreement with LC-MS measurements (Taoka et al, 2018), arguing against a systematic underestimation by SCARPET. Despite these quantitative differences, both approaches support the key conclusion that internal m⁷G occurs at appreciable stoichiometry at specific mRNA sites.

SCARPET also enabled analysis of Ψ, m^6^_2_A, and m^5^C at single-nucleotide resolution, with stoichiometries that closely matched LC-MS measurements (Taoka et al, 2018). These results illustrate that SCARPET can be used for quantitative validation of a broad set of nucleotide modifications. One exception is 2′-*O*-methylated nucleotides, which remain challenging because efficient RNase H cleavage requires an unmodified 2′-OH. Alternative guide-directed cleavage strategies, such as the use of Cas13 (Liu et al, 2024), could potentially overcome this limitation and extend the method to 2′-*O*-methylated residues.

In summary, SCARPET resolves outstanding questions about two highly debated mRNA modifications. First, SCARPET confirms that internal m^7^G is a genuine modification at specific sites in mRNA. Second, SCARPET shows that many newly reported m^1^A sites are instead inosines generated by A-to-I editing. These results emphasize the need for site-specific biochemical validation when evaluating low-abundance modifications and establish SCARPET as a robust method for this purpose. The presence of internal m^7^G at high stoichiometry raises new mechanistic and functional questions, and future studies will be needed to determine how m^7^G influences mRNA metabolism and gene expression.

## Methods

**Table:** Reagents and Tools Table

Reagent/resource	Reference or source	Identifier or catalog number
**Oligonucleotides and other sequence-based reagents**		
TLC standard Oligos	This study	Dataset EV2
SCARPET oligos	This study	Dataset EV2
RNA Enrichment probes	This study	Dataset EV2
Synthetic RNAs	This study	Dataset EV2
PrimeTime qPCR primer assay *MYC*	Integrated DNA Technologies	Hs.PT.58.26770695
PrimeTime qPCR primer assay *EIF3A*	Integrated DNA Technologies	Hs.PT.58.38355203
PrimeTime qPCR primer assay *RBM25*	Integrated DNA Technologies	Hs.PT.58.1195412
PrimeTime qPCR primer assay *ACTB*	Integrated DNA Technologies	Hs.PT.39a.22214847
PrimeTime qPCR primer assay *GAPDH*	Integrated DNA Technologies	Hs.PT.39a.22214836
PrimeTime qPCR primer assay *MALAT1*	Integrated DNA Technologies	Hs.PT.58.26451167.g
PrimeTime qPCR primer assay *18S rRNA*	Integrated DNA Technologies	Hs.PT.39a.22214856.g
**Chemicals, enzymes and other reagents**		
TRIzol reagent	Invitrogen	Cat. # 15596026
TURBO DNase	Invitrogen	Cat. # AM2239
Dynabeads™ Oligo(dT)_25_	Invitrogen	Cat. # 61005
Dynabeads^TM^ MyOne Streptavidin C1 beads	Invitrogen	Cat # 65002
SuperScript™ IV Reverse Transcriptase	Invitrogen	Cat. # 18090050
Lithium chloride solution	Sigma-Aldrich	Cat # L7026
Power SYBR Green PCR Master Mix	Applied Biosystems	Cat. # A25776
T4 polynucleotide kinase	New England Biolabs	Cat. # M0201L
[γ-³²P] ATP	PerkinElmer	Cat. # BLU002Z250UC
Phenol:chloroform:isoamyl alcohol (25:24:1, v/v)	Invitrogen	Cat. # 15593031
Nuclease P1	New England Biolabs	Cat. # M0660S
RNase H	New England Biolabs	Cat # M0297L
shrimp alkaline phosphatase (rSAP)	New England Biolabs	Cat # M0371L
Quick Ligase	New England Biolabs	Cat # M2200S
XRN-1 exonuclease	New England Biolabs	Cat # M00338S
PEI Cellulose F TLC plates	EMD Millipore	Cat # 1057250001
Pierce™ Saturated Ammonium Sulfate Solution	Thermo Scientific	Cat # 45216
Sodium Acetate (3 M), pH 5.5	Invitrogen	Cat # AM9740
Isopropanol, 99.5%	Thermo Scientific Chemicals	Cat # AC383910025
Hydrochloric acid	Sigma-Aldrich	Cat # 320331
Storage Phosphor Screen BAS-IP MS 2025 E Multipurpose Standard, 20 × 25 cm	Cytiva Life Sciences	Cat # 28956475
**Software**		
Online probe designer tool	LGC Biosearch https://www.biosearchtech.com/support/tools/design-software/chirp-probe-designer	NA
ImageQuant™ TL analysis software	Cytiva Life Sciences	
REDIportal	https://rediportal.cloud.ba.infn.it//atlas/index.html D’Addabbo et al, 2024	
**Other**		
RNA Clean & Concentrator kit	Zymo Research	Cat. # R1016
Magnetic Instant Capture (MagIC) Beads kit	ElementZero Biolabs	NA
Amersham Typhoon 5	Cytiva Life Sciences	GEH29187191EA
QuantStudio 5 Real-Time PCR System	Applied Biosystems	–

### Cell culture

HEK293T and HeLa cells were cultured in Dulbecco’s Modified Eagle Medium (DMEM) supplemented with 10% fetal bovine serum (FBS) and 1% penicillin–streptomycin. Cells were maintained at 37 °C in a humidified incubator with 5% CO₂. Cells were plated in 15 cm² dishes and harvested at ~70% confluency.

### RNA isolation and purification

Total RNA was extracted using TRIzol reagent (Invitrogen, Cat. # 15596026), following the manufacturer’s instructions. Extracted RNA was treated with TURBO DNase (Invitrogen, Cat. # AM2239) and purified using the RNA Clean & Concentrator kit (Zymo Research, Cat. # R1016). RNA concentration was measured using a NanoDrop spectrophotometer (Thermo Fisher), and samples were stored at –80 °C until further use.

For 2× poly(A)-purified mRNA, DNase-treated total RNA was used as input. Dynabeads™ Oligo(dT)_25_ (Invitrogen, Cat. # 61005) were used to perform the two rounds of purification according to the manufacturer’s instructions. Purified mRNA was quantified using NanoDrop and stored at –80 °C.

### mRNA enrichment

Magnetic Instant Capture (MagIC) Beads kit (ElementZero Biolabs) customized for human *MYC* mRNA was used according to the manufacturer’s protocol to obtain highly enriched *MYC* mRNA for comparison with the BioCapT protocol.

For the BioCapT protocol, seven nonoverlapping 5’ biotinylated DNA oligonucleotide probes were designed using the online probe designer tool from LGC Biosearch (https://www.biosearchtech.com/support/tools/design-software/chirp-probe-designer). Each oligonucleotide probe was precisely 20 nucleotides in length, with a GC content of 55%. Sequences of all the probes used here are available in Dataset EV2.

For mRNA purification, 4 μg of 2× Poly(A)-purified mRNAs was diluted with nuclease-free water to make the final volume of 230 µL. The RNA was then incubated at 75 °C for 5 min, followed by immediate transfer to ice. The RNA was then mixed with a total of 20 pmol of pooled oligonucleotides, comprising an equimolar mix of all seven oligonucleotides, in 250 µL of 2× hybridization buffer (1×: 50 mM HEPES, pH 7.5, 1% (v/v) lithium dodecyl sulfate, 500 mM lithium chloride, 5 mM EDTA, 5 mM dithiothreitol (DTT), 15% formamide) and incubated for 1 h at 48 °C with intermittent shaking (800 rpm, 30 s ON/3 min OFF). Next, 100 µg of Dynabeads^TM^ MyOne Streptavidin C1 beads (Invitrogen, Cat # 65002) equilibrated in hybridization buffer were added to the mixture and incubated at room temperature for 15 min with gentle end-to-end rotation. The samples were then placed on a magnet rack (Thermo Fisher) for 1–2 min, and supernatant was carefully pipetted out. Samples were removed from the magnetic rack and beads were resuspended gently in 400 µL wash buffer (50 mM HEPES, pH 7.5, 2% (v/v) lithium dodecyl sulfate, 500 mM lithium chloride, 5 mM EDTA, 5 mM dithiothreitol (DTT)) and incubated at 60 °C for 5 min with intermittent shaking (1000 rpm, 30 s ON/3 min OFF). The beads were again placed in a magnetic rack and the supernatant was carefully removed. Beads were washed with wash buffer three times incubating at 60 °C for 5 min each time. The beads were resuspended in elution buffer (10 mM HEPES pH 7.5, 10 mM EDTA), incubated at 92 °C for 2 min and immediately placed on the magnetic rack. The supernatant containing the eluted mRNAs was collected, quantified using the Agilent TapeStation system with the RNA High Sensitivity assay, and stored at −80 °C until use. The efficiency of the mRNA enrichment was assessed using RT-qPCR.

### RT-qPCR

Reverse transcription of RNA was carried out using SuperScript™ IV Reverse Transcriptase (Invitrogen Cat. # 18090050) in 20 µL reactions, following the manufacturer’s instructions. The cDNA was diluted to 200 µL with nuclease-free water, and 3 μL of diluted cDNA was used as the input for quantitative real-time PCR (qPCR), performed using Power SYBR Green PCR Master Mix (Applied Biosystems, Cat. # A25776) following the manufacturer’s instructions. We used predesigned PrimeTime qPCR primer assays from Integrated DNA Technologies for *MYC*, *EIF3A*, *RBM25*, *ACTB*, *GAPDH*, *18S rRNA* and *MALAT1* RNAs. The reactions were run on the QuantStudio 5 Real-Time PCR System (Applied Biosystems) and analyzed using the ΔΔCt method.

To assess mRNA enrichment efficiency, RT-qPCR data were processed as follows. First, the quantification cycle (Cq) values from each technical replicate for each RNA were transformed by the 2^^(−Cq)^ formula. Next, the transformed values obtained from every sample were divided by the values obtained for *GAPDH* or *ACTB* transcripts, thus giving a ratio of the measured transcripts in the input and enriched sample. Finally, the calculated ratios in the enriched sample were divided by the corresponding ratios obtained for the input sample, resulting in the fold enrichment value over input.

### Preparation of thin-layer chromatography standards

All TLC standards were prepared by 5′ end radiolabeling of synthetic RNA oligonucleotides using T4 polynucleotide kinase (T4 PNK, New England Biolabs, Cat. # M0201L) and [γ-³²P] ATP (PerkinElmer, Cat. # BLU002Z250UC). Each oligonucleotide, 10 nucleotides in length and containing the respective modified nucleotide at its 5′ end, was purchased from Dharmacon. Each labeling reaction contained 250 ng (50 pmol) synthetic RNA, 1 µL (10 pmol) 10 mCi/mL [γ-³²P] ATP, 1 µL (10 U) T4 PNK, and 3 μL 10X T4 PNK buffer in a final volume of 30 µL with nuclease-free water. Reactions were incubated at 37 °C for 1 h in an Eppendorf ThermoMixer C with a ThermoTop heated lid (800 rpm, 30 s ON/3 min OFF interval mixing). Labeled RNA was purified using phenol:chloroform:isoamyl alcohol (25:24:1, v/v) (Invitrogen, Cat. # 15593031), following the manufacturer’s protocol, and resuspended in 16 µL of nuclease-free water. Next, 2 µL (2 U) Nuclease P1 (New England Biolabs, Cat. # M0660S) and 2 µL 10X P1 buffer were added, and samples were incubated at 37 °C for 2 h under the same shaking conditions. Final hydrolysates were diluted to 500 µL with nuclease-free water. Sequences of all the synthetic RNA oligonucleotides used here are available in Dataset EV2.

### SCARPET assay

Sequences of all the SCARPET chimeric and self-splinting hairpin oligonucleotides used here are available in Dataset EV2. All incubation steps were performed at their specified temperatures using an Eppendorf ThermoMixer C with a ThermoTop heated lid. For each site, 1 μg of TURBO DNase-treated total RNA or 500 ng of 2X-poly(A) mRNA was mixed with ∼3 pmol of SCARPET oligonucleotide in a total volume of 3 μL of 30 mM tris-HCl, pH 7.5. For each site, a control sample was set up in which the SCARPET oligonucleotide was not added. To anneal the SCARPET oligonucleotide to the target RNA the sample was heated at 95 °C for 1 min, followed by incubation at room temperature for 3 min before putting it on ice for the next step.

#### RNase H-mediated site-specific cleavage and dephosphorylation step

For site-specific cleavage of target RNA and dephosphorylation at the 5′ end, 1 μL RNase H reaction mixture (containing 2× T4 PNK buffer, 1 U RNase H [New England Biolabs, Cat # M0297L], and 1 U (1 μL) shrimp alkaline phosphatase (rSAP, New England Biolabs, Cat # M0371L) were added to each sample. The samples were incubated at 44 °C for 1 h. The RNase H and rSAP were then heat inactivated by incubating the samples at 75 °C for 5 min and immediately put on ice.

#### Radiolabeling step

Next, 1.5 μL of T4 PNK reaction mixture (containing 1X T4 PNK buffer, 6 U of T4 PNK) and 1 μL of 10 μCi/μL [γ-32P]-ATP was added to each sample, making a total volume of 7.5 μL. The samples were incubated at 37 °C for 1 h to radiolabel the cleaved target RNA at its 5′ end. T4 PNK was heat inactivated by incubating the samples at 75 °C for 5 min and immediately put on ice.

#### Self-splinting hairpin-assisted ligation

For self-splinting hairpin oligonucleotide-assisted ligation, 2.5 μL of 10 μM (25 pmol) of a 5′-biotinylated self-splinting hairpin oligonucleotide (5′Bio-ssHP) was added to each sample, and samples were mixed using a pipette. For proper folding of 5′Bio-ssHP and splint annealing to the radiolabeled target RNA, samples were heated at 75 °C for 3 min followed by incubation at room temperature for 3 min, and then samples were put on ice. Next, 10 μL of 2× Quick Ligase buffer [New England Biolabs, Cat # M2200S] and 1 μL of Quick Ligase enzyme was added to each sample, and samples were incubated at 25 °C for 1 h. The target RNA ligated to the 5′Bio-ssHP was captured by incubation of the solution to MyOne Streptavidin C1 magnetic beads. The beads were prepared according to the manufacturer’s instructions for RNA applications. To each sample, 4 μL of nuclease-free water was added to bring up the final volume to 25 μL. Next, 25 μL of MyOne Streptavidin C1 beads (75 μg beads in 2× B&W buffer [10 mM Tris-HCl, pH 7.5, 1 mM EDTA, and 2 M NaCl]) was added to each sample bringing the total volume to 50 μL. Samples were incubated at room temperature with end-to-end rotations for 15 min for efficient coupling. Next, samples were put back on a magnet rack (Thermo Fisher) for 1–2 min, and liquid was carefully pipetted out into radioactive liquid waste. Samples were removed from the magnetic rack and beads were washed first with 50 μL of 1× B&W buffer and tubes were placed back on the magnetic rack for 1–2 min, and supernatant was carefully removed. The second wash was performed like the first one with 50 μL of 10 mM Tris-HCl pH 7.5.

#### Exonuclease step

Next, 20 μL of XRN-1 exonuclease [New England Biolabs, Cat # M00338S] reaction mixture (containing 2 μL of NEB Buffer 3 (10X), 1 μL XRN-1, and 17 μL of nuclease-free water) was added to each sample and incubated at 37 °C for 1 h. Samples were put back on a magnetic rack for 1–2 min, and supernatant was carefully removed. Next, samples were subjected to two rounds of washes as described before.

#### RNase H step

For the RNase H step, 10 μL of RNase H reaction mixture (containing 1 μL 10X T4 PNK buffer, 5 U [1 μL] RNase H, 8 μL of nuclease-free water) was added to each sample, mixed with a pipette, and incubated at 37 °C for 1 h.

#### Nuclease P1 step

Finally, samples were digested to 5′-monophosphate nucleotides by adding 3.5 U (3.5 μL) of nuclease P1 and 1.5 μL of 10X nuclease P1 buffer (New England Biolabs, Cat # M0660S) and incubated at 37 °C for 1 h. Next, samples were placed on a magnetic rack for 1–2 min, and the hydrolysates were transferred to fresh tubes.

#### Thin-layer chromatography (TLC) step

To detect the modified residues, the nucleotide hydrolysate from each sample was separated by 1D TLC on precoated PEI Cellulose F TLC plates (EMD Millipore, Cat # 1057250001) as described previously (Grosjean et al, 2004). Briefly, 1 μL RNA hydrolysate from each sample was spotted alongside the respective RNA nucleotide TLC standard. After the sample spots dried, the TLC plate was placed in a TLC developing chamber with 100 mL of respective solvent buffer and allowed to run. For Solvent A: isopropanol:concentrated HCl:water [70:15:15, v/v/v], the TLC plate was allowed to develop overnight (∼14 h). For Solvent B: Saturated (NH_4_)_2_SO_4_:10 mM sodium acetate, pH 6.0:isopropanol [79∶19∶2, v/v/v] the run was for ~5 h. Next, TLC plates were dried at room temperature for 1 h, wrapped with SARAN wrap, and exposed to BAS storage phosphor screens (Cytiva Life Sciences) for 6–12 h. The phosphor screens were imaged using an Amersham Typhoon 5 (Cytiva Life Sciences). The phosphor screen images were analyzed using ImageQuant™ TL analysis software.

### Reanalysis of putative m¹A sites

Putative internal m¹A sites reported by Zhou et al, (2019) were reanalyzed using processed m¹A-quant-seq data from the Gene Expression Omnibus (GEO accession GSE123365) and the reanalysis framework described by Wiener and Schwartz (Wiener and Schwartz, 2021). For each site, A → G, A → C, and A → T mutation counts were tabulated, and the predominant mutation type was determined. Sites exhibiting predominantly A → G mutations were cross-referenced against the REDIportal (D’Addabbo et al, 2024) and DARNED (Kiran et al, 2013) databases to identify known A-to-I RNA editing events. Sites not classified as A-to-I editing were assessed for the presence of known genomic variants. Genomic coordinates originally reported in the hg19 reference genome were converted to GRCh38.p14 using Ensembl BioMart, and the updated coordinates were queried against human genetic variation data from the Ensembl Genes 115 database, in accordance with methods described by Kinsella et al (Kinsella et al, 2011). Variant consequences were annotated via Ensembl, and somatic variants were identified through the COSMIC database. The complete reanalysis, including mutation counts, editing annotations, variant classifications, and GRCh38 coordinates, is provided as Dataset EV1.

## Supplementary information

Peer Review File

Dataset EV1

Dataset EV2

Source data Fig. 1

Source data Fig. 2

Source data Fig. 3

Source data Fig. 4

Source data Fig. 5

Source data Fig. 6

All EV Figures combined Source Data

Expanded View Figures

## Data Availability

The m¹A-quant-seq data reanalyzed in this study are publicly available in the Gene Expression Omnibus (GEO) under accession number GSE123365 (Zhou et al, 2019). The source data of this paper are collected in the following database record: biostudies:S-SCDT-10_1038-S44319-026-00840-2.
