# Supplementary material for: Validation of the mRNA epitranscriptome: SCARPET reveals that mapped m1A sites are inosine
Source: EMBO Rep. 2026 Jun 23;27(15):4357–75. doi: 10.1038/s44319-026-00840-2 (PMC13458256; doi:10.1038/s44319-026-00840-2)
Supplement: Supplementary file 1 [file 44319_2026_840_MOESM11_ESM.pdf]

Expanded View Figures

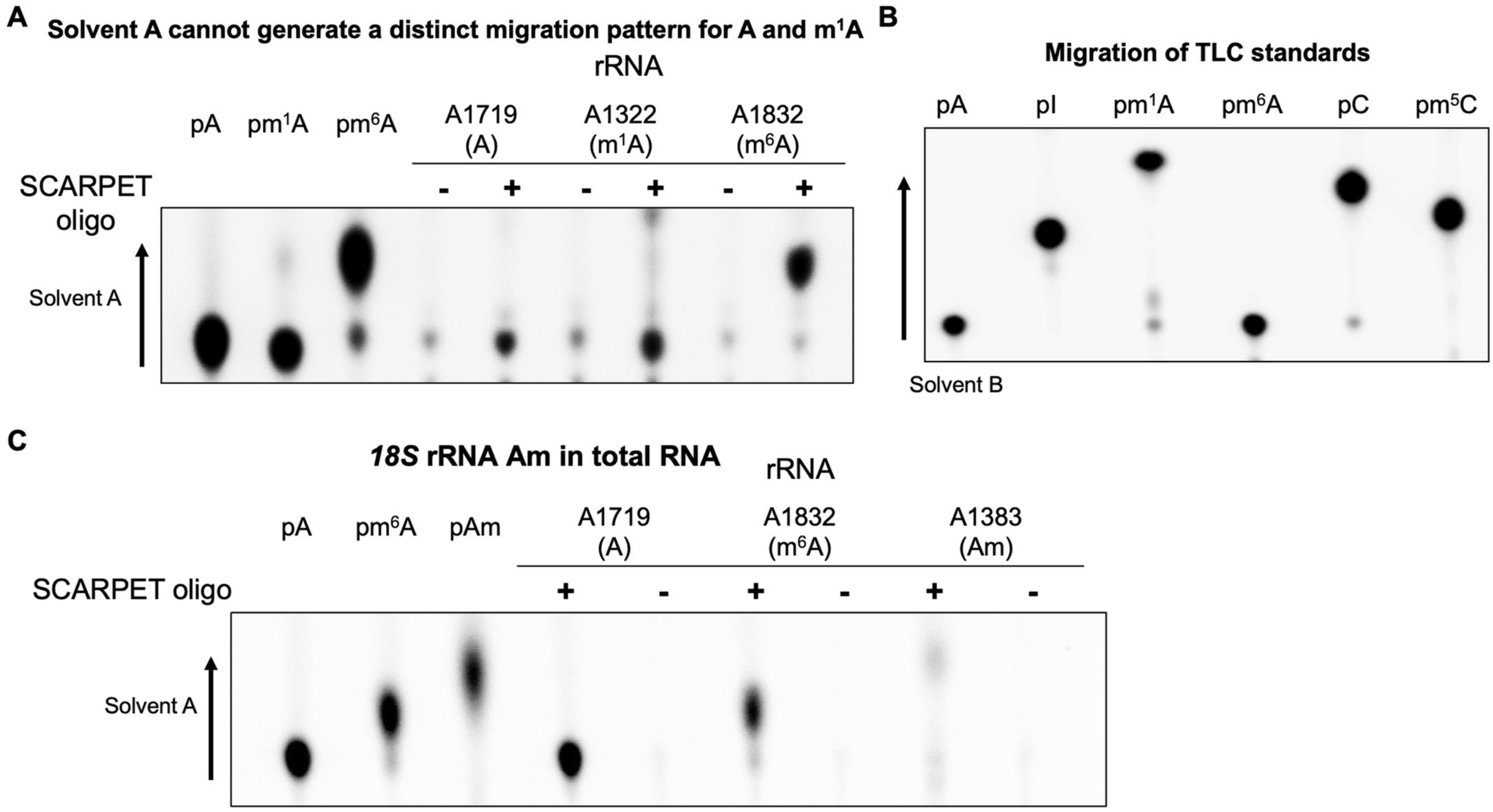

Figure EV1. Solvent optimization improves resolution of RNA modifications by SCARPET.

(**A**) SCARPET analysis using solvent A (Isopropanol: Concentrated HCl: Water, 70:15:15) does not generate distinct TLC migration profiles for adenosine (pA) and 1-methyladenosine (pm1A). (**B**) TLC separation of nucleotide standards using solvent B (saturated (ammonium sulfate:10 mM sodium acetate, pH 6.0: Isopropanol, 79:19:2) demonstrates improved resolution for Inosine (pI), pm1A, and pm5C, which migrate distinctly from their unmodified counterparts. (**C**) SCARPET analysis of residue A1383 in 18S rRNA shows a very faint signal at the Am position, indicating that SCARPET is largely ineffective at detecting 2′-O-methyl modifications.

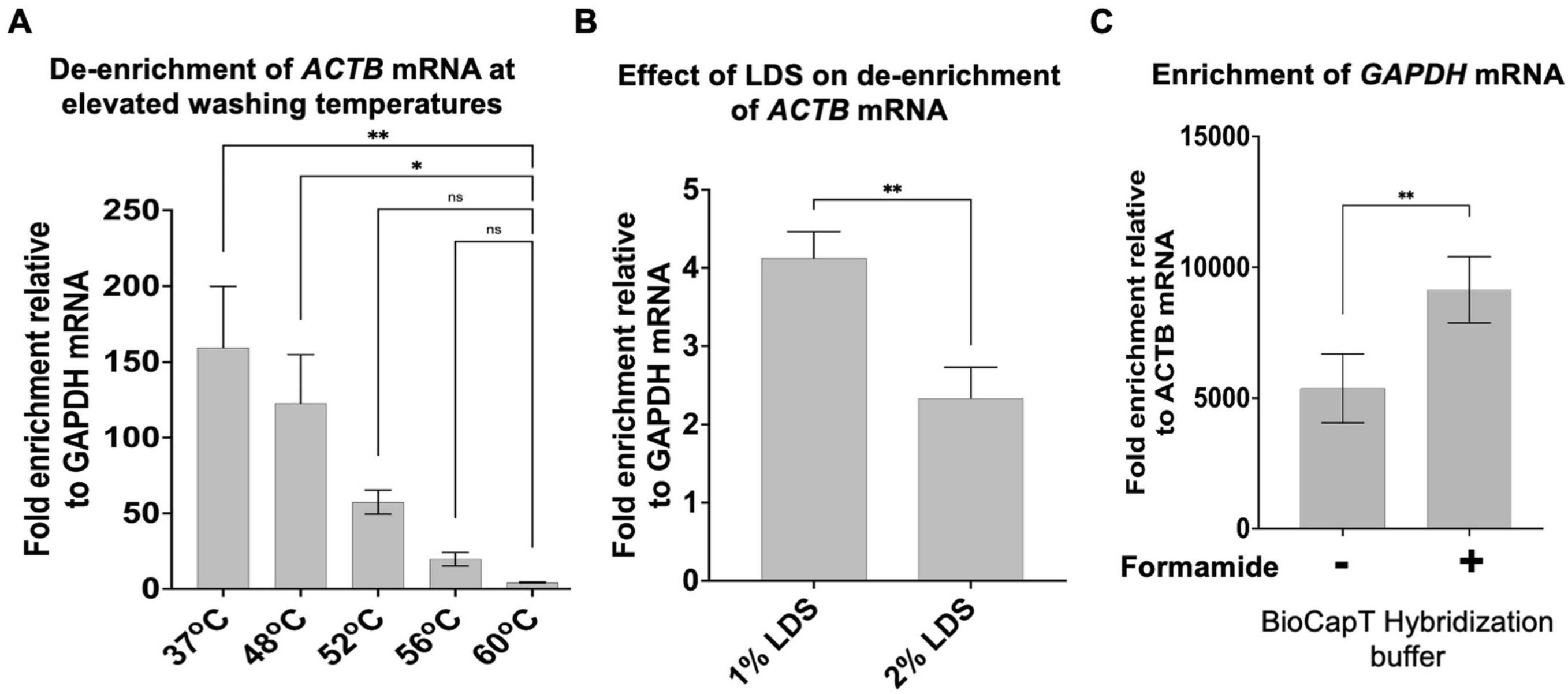

Figure EV2. Optimization of washing stringency enhances transcript-specific RNA enrichment by reducing non-specific background.

(**A**) Relative levels of non-target *ACTB* mRNA compared with *GAPDH* mRNA decreased with elevated washing temperatures in BioCapT protocol for *MYC* mRNA enrichment. Error bars indicate SD. Data represent mean ± SD from three independent biological replicates ($N = 3$). Statistical significance was determined using ordinary one-way ANOVA followed by Tukey's multiple comparisons test. Adjusted P values: 60 °C vs 37 °C, $P < 0.0001$; 60 °C vs 48 °C, $P = 0.0003$; 60 °C vs 52 °C, $P = 0.0096$; 60 °C vs 56 °C, $P = 0.8382$ (ns). Significance levels: *$P < 0.05$, **$P < 0.01$, ***$P < 0.001$, ****$P < 0.0001$; ns = not significant. (**B**) Increasing the LDS concentration from 1% to 2% during wash steps significantly reduced *ACTB* levels (**$P < 0.01$, unpaired t test) in BioCapT enriched *MYC* mRNA. Error bars indicate SD. Data represent mean ± SD from three independent biological replicates ($N = 3$). (**C**) Enrichment of *GAPDH* mRNA relative to *ACTB* mRNA using BioCapT protocol for *GAPDH* mRNA enrichment (**$P < 0.01$, unpaired t test). Error bars indicate SD. Data represent mean ± SD from three independent biological replicates ($N = 3$).

 © The Author(s)

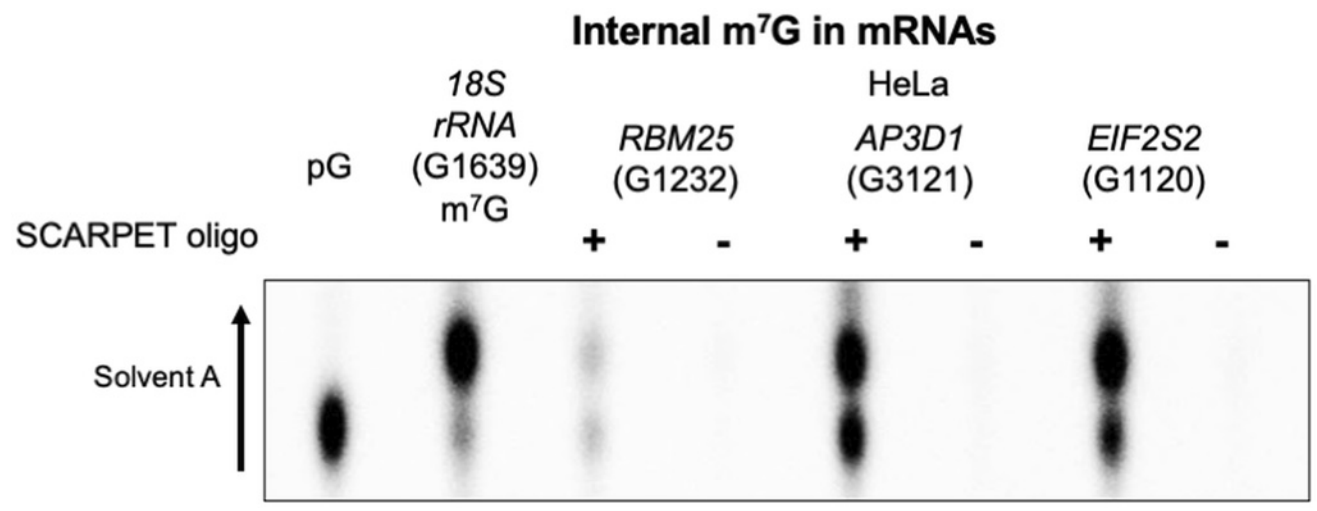

Figure EV3. SCARPET detects internal m⁷G in *RBM25*, *AP3D1*, and *EIF2S2* mRNAs.

SCARPET validation of candidate internal m^{7}G sites in *RBM25* (G1232), *AP3D1* (G3121), and *EIF2S2* (G1120) mRNAs in HeLa cells. G1639 in *18S* rRNA served as a positive control for internal m^{7}G ($N = 2$).
