# Supplementary material for: Validation of the mRNA epitranscriptome: SCARPET reveals that mapped m1A sites are inosine
Source: EMBO Rep. 2026 Jun 23;27(15):4357–75. doi: 10.1038/s44319-026-00840-2 (PMC13458256; doi:10.1038/s44319-026-00840-2)
Supplement: Supplementary file 2 [file 44319_2026_840_MOESM1_ESM.pdf]

EMBO reports

Validation of the mRNA epitranscriptome: SCARPET reveals that mapped m1A sites are inosine

Rohit Nalavade, Aashiq Mirza, and Samie Jaffrey

Corresponding author(s): Samie Jaffrey (srj2003@med.cornell.edu)

Review Timeline:

Submission Date:	25th Nov 25
Editorial Decision:	8th Jan 26
Revision Received:	18th Apr 26
Editorial Decision:	11th May 26
Revision Received:	21st May 26
Accepted:	8th Jun 26

Editor: Esther Schnapp

Transaction Report:

(Note: With the exception of the correction of typographical or spelling errors that could be a source of ambiguity, letters and reports are not edited. Depending on transfer agreements, referee reports obtained elsewhere may or may not be included in this compilation. Referee reports are anonymous unless the Referee chooses to sign their reports.)

1st Editorial Decision 8th Jan 2026

Dear Samie, and happy new year!

Thank you for the submission of your manuscript to EMBO reports. We have now received the full set of referee reports that is pasted below.

As you will see, all referees acknowledge that the findings are interesting. However, they also have some suggestions for how the study could be improved and strengthened. I think all suggestions are good and should be addressed, but please let me know in case you disagree and we can discuss the exact revision requirements further, also in a video chat, if you like.

I would thus like to invite you to revise your manuscript with the understanding that the referee concerns must be fully addressed and their suggestions taken on board. Please address all referee concerns in a complete point-by-point response. Acceptance of the manuscript will depend on a positive outcome of a second round of review. It is EMBO reports policy to allow a single round of major revision only and acceptance or rejection of the manuscript will therefore depend on the completeness of your responses included in the next, final version of the manuscript.

We realize that it is difficult to revise to a specific deadline. In the interest of protecting the conceptual advance provided by the work, we recommend a revision within 3 months (10th Apr 2026). Please discuss the revision progress ahead of this time with the editor if you require more time to complete the revisions.

You can either publish the study as a short report or as a full article. For short reports, the revised manuscript should not exceed 27,000 characters (including spaces but excluding materials & methods and references) and 5 main plus 5 expanded view figures. The results and discussion sections must further be combined, which will help to shorten the manuscript text by eliminating some redundancy that is inevitable when discussing the same experiments twice. For a normal article there are no length limitations, but it should have more than 5 main figures and the results and discussion sections must be separate. In both cases, the entire materials and methods must be included in the main manuscript file.

IMPORTANT NOTE: we perform an initial quality control of all revised manuscripts before re-review. Your manuscript will FAIL this control and the handling will be DELAYED if the following APPLIES:
1) A data availability section providing access to data deposited in public databases is missing. If you have not deposited any data, please add a sentence to the data availability section that explains that.
2) Your manuscript contains statistics and error bars based on n=2. Please use scatter blots in these cases. No statistics should be calculated if n=2.

When submitting your revised manuscript, please review the submission guidelines (https://link.springer.com/journal/44319/submission-guidelines#cms-Revised-submissions) and carefully review the instructions that follow below. Failure to include requested items will delay the evaluation of your revision.

1) a .docx formatted version of the manuscript text (including legends for main figures, EV figures and tables). Please make sure that the changes are highlighted to be clearly visible.

2) individual production quality figure files as .eps, .tif, .jpg (one file per figure). See hhttps://media.springernature.com/original/springer-cms/rest/v1/content/27825798/data/v1 for more info on how to prepare your figures.

3) We replaced Supplementary Information with Expanded View (EV) Figures and Tables that are collapsible/expandable online. A maximum of 5 EV Figures can be typeset. EV Figures should be cited as 'Figure EV1, Figure EV2" etc... in the text and their respective legends should be included in the main text after the legends of regular figures.

- For the figures that you do NOT wish to display as Expanded View figures, they should be bundled together with their legends in a single PDF file called *Appendix*, which should start with a short Table of Content. Appendix figures should be referred to in the main text as: "Appendix Figure S1, Appendix Figure S2" etc. See 'Expanded View' section of the submission guidelines.

- Additional Tables/Datasets should be labeled and referred to as Table EV1, Dataset EV1, etc. Legends have to be provided in a separate tab in case of .xls files. Alternatively, the legend can be supplied as a separate text file (README) and zipped together with the Table/Dataset file.

4) a .docx formatted letter INCLUDING the reviewers' reports and your detailed point-by-point responses to their comments. As part of the EMBO Press transparent editorial process, the point-by-point response is part of the Review Process File (RPF), which will be published alongside your paper.

5) a complete author checklist, which you can download from our author guidelines. Please insert information in the checklist that is also reflected in the manuscript. The completed author checklist will also be part of the RPF.

6) Please note that all corresponding authors are required to supply an ORCID ID for their name upon submission of a revised manuscript (), which can be linked to your profile in the manuscript submission system.

7) Before submitting your revision, primary datasets produced in this study need to be deposited in an appropriate public database. Please remember to provide a reviewer password if the datasets are not yet public. The accession numbers and database should be listed in a formal "Data Availability" section placed after Methods. Please note that the Data Availability Section is restricted to new primary data that are part of this study. * Note - All links should resolve to a page where the data can be accessed. *

If your study has not produced novel datasets, please mention this fact in the Data Availability Section.

8) At EMBO Press we ask authors to provide source data for the main manuscript figures. You will receive a separate email with instructions for providing source data with your revised manuscript, including how to upload and organize the files.

9) Our journal also encourages inclusion of *data citations in the reference list* to directly cite datasets that were re-used and obtained from public databases. Data citations in the article text are distinct from normal bibliographical citations and should directly link to the database records from which the data can be accessed. In the main text, data citations are formatted as follows: "Data ref: Smith et al, 2001" or "Data ref: NCBI Sequence Read Archive PRJNA342805, 2017". In the Reference list, data citations must be labeled with "[DATASET]". A data reference must provide the database name, accession number/identifiers and a resolvable link to the landing page from which the data can be accessed at the end of the reference.

10) Regarding data quantification, the following points must be specified in each figure legend:

- the name of the statistical test used to generate error bars and P values,

- the number (n) of independent experiments (please specify technical or biological replicates) underlying each data point,

- the nature of the bars and error bars (s.d., s.e.m.),

- If the data are obtained from n Program fragment delivered error ``Can't locate object method "less" via package "than" (perhaps you forgot to load "than"?) at //ejpvfs23/sites23b/embor_www/letters/embor_decision_revise_and_review_sr_any.txt line 55.' 2, use scatter blots showing the individual data points.

Discussion of statistical methodology can be reported in the materials and methods section, but figure legends should contain a basic description of n, P and the test applied.

- Please also include scale bars in all microscopy images.

11) The journal requires a statement specifying whether or not authors have competing interests (defined as all potential or actual interests that could be perceived to influence the presentation or interpretation of an article). In case of competing interests, this must be specified in your disclosure statement.

12) All Materials and Methods need to be described in the main text using our 'Structured Methods' format, which is required for all research articles. According to this format, the Methods section includes a separate Reagents and Tools Table file (listing key reagents, experimental models, software and relevant equipment and including their sources and relevant identifiers) and a Methods and Protocols section describing the methods using a step-by-step protocol format. The aim is to facilitate adoption of the methodologies across labs. More information on how to adhere to this format as well as a downloadable template (.docx) for the Reagents and Tools Table can be found in our author guidelines.

We would also welcome the submission of cover suggestions, or motifs to be used by our Graphics Illustrator in designing a cover.

As part of the EMBO publication's Transparent Editorial Process, EMBO reports publishes online a Review Process File (RPF) to accompany accepted manuscripts. This File will be published in conjunction with your paper and will include the referee reports, your point-by-point response and all pertinent correspondence relating to the manuscript.

You are able to opt out of this by letting the editorial office know (emboreports@embo.org). If you do opt out, the Review Process File link will point to the following statement: "No Review Process File is available with this article, as the authors have chosen not to make the review process public in this case."

I look forward to seeing a revised form of your manuscript when it is ready.

Best wishes,
Esther

Esther Schnapp, PhD
Senior Editor
EMBO reports

Referee #1:

In this manuscript, Nalavade et al apply SCARPET in order to validate the presence of two controversial modifications on mRNA: m1A and m7G. Both modifications had been reported to be widespread, yet the findings have been controversial. SCARPET had been previously developed and employed by the authors in the context of m6A analysis, and this manuscript now sets out to apply it in the context of additional modifications, concluding that m7G sites can be confirmed whereas m1A sites had been erroneously called and in fact originated from inosine.
The manuscript is well-motivated and well-written, and the community is in need of clarity in terms of which modifications are present versus absent on mRNA. As such we are therefore generally supportive of these findings.
Nonetheless, there are two points which should be weighed, both drawing on a Wiener et al Nature Reviews Genetics paper, 2021(cited in this manuscript). In this paper, two sets of analyses are shown, precisely concerning the two modifications here. Regarding m1A, this paper reanalyzes the same dataset analyzed here, and arrives at an identical conclusion, namely that the called sites are attributed to inosine, rather than to m1A. This conclusion relies on similar considerations as are listed in the current manuscript, namely its distribution in dsRNAs, the A->G (rather than A->T) mutational profile, and the fact that the identified sites are comprised in editing databases. While these consistent conclusions should be better acknowledged, we emphasize that they should not undermine the publication of the current paper - which relies on a firm set of biochemical measurements, rather than on conjecture.
The second point pertains to m7G. In the above-listed paper the authors had also reanalyzed the entirety of the m7G datasets provided by the Zhang et al m7G study. This study had relied on identifying mismatches following a chemical treatment specific to m7G. However, the NRG manuscript found that such mismatches could not be found at nearly any of the sites once an aligner procedure was employed that allows soft-clips (previously shown to be crucial for such mutational studies). This is true also for all of the sites that now appear to be confirmed by the authors - none of them, with the possible exception of EIF2S2, shown evidence of mutational signatures. This does not, of course, prove that there is no m7G at the sites, and yet it raises critical questions regarding whether there had been any basis for having identified these sites as putatively modified in the first place. Needless to say, should the authors firmly believe that these sites are now robustly validated they have every right to publish them as such, and we would certainly not want to stand in their way. However, we would encourage the authors to perform two things: (1) We would strongly suggest that the authors download the published dataset in the Zhang et al study themselves, and form their independent conclusions, and (2) We would strongly urge the authors to select some negative controls (perhaps from within the same genes, that can be enriched using the similar approach), and ensure that these do not give rise to m7G signatures. While the consistent signal observed at every single one of the tested targets could be consistent with every one of them being methylated to a sizeable extent, it could also be consistent with a consistent artifact (perhaps labelling and contamination by naturally occurring m7G caps?) impacting the measurements.

Referee #2:

The need for orthogonal validation of reported modification sites is a major issue in the field of epitranscriptomics. In this context, the manuscript of Nalavade et al. here employ their recently introduced method, SCARPET (PMID: 38190635), to validate RNA modifications other than m6A, which was the focus of their first study. Essentially, they here improved their protocol and extend the usability of SCARPET to other RNA modifications. The authors show that SCARPET can validate a variety of RNA modifications, including but not limited to, m1A, m6A, I, ψ and m7G. Using SCARPET, the authors asked whether previously identified m1A and m7G sites in epitranscriptome are bona fide modifications sites or not. They show that previously reported m1A sites, contain inosine, as already reported by Wiener & Schwartz, 2021. Moreover, SCARPET is able to validate previously reported m7G sites. Overall, the manuscript is easy to read and corroborates the great potential of SCARPET as a method for orthogonally validating RNA modification mapping. In that sense, this is mostly a methodology paper with little new biology, which is fine. However, its widespread adoption may be limited, because SCARPET relies on radioactive labeling, which is increasingly discouraged. A main concern is whether SCARPET is applicable to sites with medium to low stoichiometry. In addition, some of the interpretations of the provided data seem to be overinterpretations.
Major Points
1. In Fig. 1., the authors tested the ability of SCARPET to detect modifications on sites with high stoichiometry (90-100%). However, it is not clear from the provided data that SCARPET can detect sites with lower stoichiometry. As many modification sites do not have 90-100% stoichiometry, whether SCARPET can or cannot detect such sites is a critical question. Therefore, the authors should demonstrate the limit of detection of SCARPET and if it differs from modification to modification. One way to demonstrate this could be to do a titration of synthetic RNAs with 100% modified site and mixing it with unmodified RNA, in different rations (10%, 20, 30,40%...) and monitor if they can detect modifications. Another experiment to this end could be testing sites with lower stoichiometry from cellular RNA with SCARPET and an orthogonal approach.

2. In all SCARPET analysis done in the study, only singlet samples without replicate were provided. For confident quantification of stoichiometry and to evaluate the precision of SCARPET, the authors should include additional replicates to strengthen the reliability of the results, especially in Fig 3.
3. The authors state that: "Based on the biochemical analysis, our data appears to resolve the m1A issue and confirms that the only sites with readily detectable m1A stoichiometry in HEK293T cells are PRUNE, ND5 and the MALAT1 ncRNA, as described previously" seems to be an overinterpretation since only ND5 is tested by SCARPET in the study. The authors should also test PRUNE and MALAT1 with SCARPET to support this claim.
4. The finding that m1A sites, identified by Zhou et al., are, to a large extent, likely representing inosine is of limited novelty because Wiener& Schwart, 2021 have already demonstrated it. Adding to their finding, only three sites are validated by SCARPET in the manuscript.
5. The data supporting the following statement: "Among the other m 1 A sites identified by Zhou et al., 19 were identified as inosine in either REDIportal or DARNED. Most of the remaining sites correspond to known genomic SNPs." is missing in the manuscript. The authors should either provide these data or delete it from the manuscript.
6. In Fig. 1B, and in the rest of the manuscript, a reference standard for m7G is missing. They should include the m7G reference standard in SCARPET analysis. If they are unable to do so, they should at least clearly demonstrate that in their hands, G1639 in 18S rRNA has also almost 100% stoichiometry by an orthogonal approach.

Minor Points
1. The authors state that "The 3′-deoxyribonucleotide pairs with the nucleotide immediately upstream of the target site and directs RNase H to cleave at the 5′ side of the target residue.". However, in Fig. 1A, 5'-deoxyribonucleotide pairs with the nucleotide immediately upstream of the target site. The authors should correct this discrepancy between the figure and the text.
2. In Fig. 1C and all other tables displaying stoichiometry quantifications, the study from which the reference value was taken should be cited.
3. In Fig. 3, the authors measure the stoichiometry of previously identified m7G site by (Zhang et al., 2019) significantly lower than reported by Zhang et al. The authors should discuss this difference. Is this simply because of different labs having different batches of HEK293T that show variability on these sites, or is it because SCARPET underestimates the stoichiometry of the given sites? If possible, the authors should validate the SCARPET analysis of these sites with an orthogonal approach themselves.
4. In Fig. S2, the authors show the depletion of non-target mRNA ACTB over GAPDH mRNA. However, in the corresponding Figure legend and the text, it is not clear that the target of their enrichment is GAPDH mRNA. This creates confusion for the reader, considering that in Fig. 2, GAPDH mRNA is rather depleted as the target of BioCapT RNA enrichment in this figure is MYC mRNA. The authors should state the target mRNA in Fig. S2 more clearly.
5. The authors state that "... a reverse transcriptase that causes highly efficient A-to-U misincorporations upon encountering an m1A residue (Zhou et al., 2019)" appears to be scientifically incorrect. The reverse transcriptase rather causes an A-to-T mutation. Please revise accordingly.
6. I recommend rephrasing the following sentences for clarity:
a. "To test this, we performed SCARPET on A1322, the known in 28S rRNA, which is a known m A site that is modified at nearly 100% stoichiometry"
b. "When poly(A) RNA was used, the major signal was was from the adenosine spot, with a faint m 6 A signal"
c. "In this study the internal m 7 G sites were based on the sites that were found in common two orthogonal techniques..."

Referee #3:

RNA modifications play many important roles in gene and cell regulation. While a handful of the over 170 RNA modifications have been well characterized and established as bona fide mRNA modifications, others such as m1A and m7G have been controversial. The initial discovery of these modifications within mRNA have mostly been done by sequencing-based techniques and an orthogonal detection method has mostly (if not completely) not been utilized to validate the presence of these modifications within mRNA. Here, Nalavade et al. adapted their previous method SCARPET for m6A detection to instead detect other potential mRNA modifications like m1A, m7G, pseudouridine, m6,6A and m5C.

The results are conclusive in demonstrating that SCARPET can biochemically detect and quantify the aforementioned RNA modifications. Using SCARPET, Nalavade et al. have clearly shown that m7G is present internally within mRNA while m1A is only present in only a few mRNAs. This manuscript has provided a much-needed validation in support of m7G as an internal mRNA modification and more importantly, clarifying the m1A controversy and showing that m1A is not a widespread mRNA modification.

While not necessary for this manuscript, the authors ought to consider using SCARPET to clear up the similar controversy of ac4C being a widespread internal mRNA modification. Besides this, my only concern is that for figures 1b, 3b, 3d, 3g and S3: Where is the pm7G standard? This should be present in the TLC images. If not, is there a reason why the authors are not able to include a pm7G standard?

Regardless, I highly recommend this manuscript for publication in EMBO Reports.

Point-by-Point Response to Reviewer Comments

We would like to thank the reviewers for their insightful comments and constructive suggestions. All the reviewers were positive about our study. Referee #1 described the manuscript as "well-motivated and well-written" and noted that "the community is in need of clarity in terms of which modifications are present versus absent on mRNA", adding that they are "generally supportive of these findings". Referee #2 noted that "the manuscript is easy to read and corroborates the great potential of SCARPET as a method for orthogonally validating RNA modification mapping". Referee #3 stated that "the results are conclusive in demonstrating that SCARPET can biochemically detect and quantify the aforementioned RNA modifications", and that "this manuscript has provided a much-needed validation in support of m^7G as an internal mRNA modification and more importantly, clarifying the m^1A controversy". We greatly appreciate the enthusiasm all the reviewers showed for our manuscript.

We have performed all the suggested experiments and addressed every question raised by the reviewers. As a result of these changes, the revised manuscript is substantially strengthened with new data demonstrating the quantitative accuracy of SCARPET across a broad stoichiometric range, direct biochemical validation of m^1A at the three previously established sites, appropriate negative controls for m^7G, and a comprehensive re-annotation of all candidate m^1A sites from Zhou et al. (2019).

We now present a revised manuscript in which each of the reviewers' individual comments is addressed as follows:

Referee #1:

Referee #1 noted that "the community is in need of clarity in terms of which modifications are present versus absent on mRNA". The reviewer raised two important points drawing on the prior computational reanalysis by Wiener & Schwartz (2021) of the m^1A and m^7G datasets and suggested including negative controls within the same enriched transcripts. We have addressed both points as described below.

In this manuscript, Nalavade et al apply SCARPET in order to validate the presence of two controversial modifications on mRNA: m1A and m7G. Both modifications had been reported to be widespread, yet the findings have been controversial. SCARPET had been previously developed and employed by the authors in the context of m6A analysis, and this manuscript now sets out to apply it in the context of additional modifications, concluding that m7G sites can be confirmed whereas m1A sites had been erroneously called and in fact originated from inosine.

The manuscript is well-motivated and well-written, and the community is in need of clarity in terms of which modifications are present versus absent on mRNA. As such we are therefore generally supportive of these findings.

Nonetheless, there are two points which should be weighed, both drawing on a Wiener et al Nature Reviews Genetics paper, 2021(cited in this manuscript). In this paper, two sets of analyses are shown, precisely concerning the two modifications here. Regarding m1A, this paper reanalyzes the same dataset analyzed here, and arrives at an identical conclusion, namely that the called sites are attributed to inosine, rather than to m1A. This conclusion relies on similar considerations as are listed in the current manuscript, namely its distribution in dsRNAs, the A->G (rather than A->T) mutational profile, and the fact that the identified sites are

comprised in editing databases. While these consistent conclusions should be better acknowledged, we emphasize that they should not undermine the publication of the current paper - which relies on a firm set of biochemical measurements, rather than on conjecture. The second point pertains to m7G. In the above-listed paper the authors had also reanalyzed the entirety of the m7G datasets provided by the Zhang et al m7G study. This study had relied on identifying mismatches following a chemical treatment specific to m7G. However, the NRG manuscript found that such mismatches could not be found at nearly any of the sites once an aligner procedure was employed that allows soft-clips (previously shown to be crucial for such mutational studies). This is true also for all of the sites that now appear to be confirmed by the authors - none of them, with the possible exception of EIF2S2, shown evidence of mutational signatures. This does not, of course, prove that there is no m7G at the sites, and yet it raises critical questions regarding whether there had been any basis for having identified these sites as putatively modified in the first place. Needless to say, should the authors firmly believe that these sites are now robustly validated they have every right to publish them as such, and we would certainly not want to stand in their way. However, we would encourage the authors to perform two things: (1) We would strongly suggest that the authors download the published dataset in the Zhang et al study themselves, and form their independent conclusions, and (2) We would strongly urge the authors to select some negative controls (perhaps from within the same genes, that can be enriched using the similar approach), and ensure that these do not give rise to m7G signatures. While the consistent signal observed at every single one of the tested targets could be consistent with every one of them being methylated to a sizeable extent, it could also be consistent with a consistent artifact (perhaps labelling and contamination by naturally occurring m7G caps?) impacting the measurements.

Response: We thank the reviewer for the generous and thoughtful assessment and for raising these important points with such clarity.

Regarding m^1A and the Wiener & Schwartz (2021) analysis:
We fully agree that Wiener & Schwartz (2021) independently reached the same conclusion about the misannotation of m^1A sites as inosine, based on computational reanalysis of the Zhou et al. dataset. This precedent deserves clearer acknowledgment than was given in the original manuscript. In the revised manuscript, we have strengthened this attribution throughout, making explicit that our computational observations, the A-to-G mutational profiles, the overlap with A-to-I editing databases, and the genomic variants are fully consistent with, and build upon, the prior bioinformatic inference of Wiener & Schwartz (2021). We emphasize, as the reviewer kindly notes, that the key contribution of the present study is to provide the first direct, single-nucleotide resolution biochemical confirmation of these conclusions.

Regarding m^7G and the soft-clip reanalysis:
We thank the reviewer for raising this critical point. We acknowledge that the reanalysis by Wiener & Schwartz (2021), showing that m^7G-associated mutational signatures in the Zhang et al. dataset largely disappear when an aligner permitting soft-clips is employed, raises legitimate questions about the computational evidence underlying those sites. We agree that an independent computational reanalysis of the Zhang et al. sequencing data would be informative; however, we believe such an analysis is beyond the scope and technical focus of the current manuscript, which is centered on biochemical rather than computational validation.

Critically, SCARPET provides evidence that is entirely independent of alignment algorithms, mismatch-calling strategies, or soft-clip handling. The method directly detects the physical presence of a modified nucleotide through its TLC migration behavior, and its validity is therefore not affected by the computational concerns raised regarding the Zhang et al. dataset.

Regarding the reviewer's suggestion to include negative controls within the same enriched transcripts to rule out systematic artifacts: We agree that this is a very good control. To address the reviewer's suggestion, we performed SCARPET on guanosine residues immediately adjacent to the putative m^7G sites, within the same transcripts (Fig. 4D, 4G). Both controls produced a single spot co-migrating exclusively with unmodified G, with no detectable m^7G signal. This directly addresses the cap contamination concern. The complete absence of m^7G signal at the neighboring sites, combined with the robust and reproducible m^7G signal at the putative sites, supports the idea that the observed modifications are site-specific and not attributable to a systematic artifact.

We are grateful to Referee #1 for the supportive assessment of our work and thoughtful suggestions. The suggestion to include negative controls within the same enriched transcripts was particularly valuable, as it directly addresses the cap-contamination concern and provides some of the strongest evidence for the site-specificity of internal m^7G.

Referee #2:

Referee #2 recognized the importance of SCARPET-based validation in the field of epitranscriptomics and provided several constructive suggestions for strengthening the manuscript. These suggestions included demonstrating the limit of detection of SCARPET, extending the validation to additional m^1A sites, and providing supporting data for the re-annotation of m^1A sites. We have addressed each of these suggestions with new experiments and data as described below:

The need for orthogonal validation of reported modification sites is a major issue in the field of epitranscriptomics. In this context, the manuscript of Nalavade et al. here employ their recently introduced method, SCARPET (PMID: 38190635), to validate RNA modifications other than m6A, which was the focus of their first study. Essentially, they here improved their protocol and extend the usability of SCARPET to other RNA modifications. The authors show that SCARPET can validate a variety of RNA modifications, including but not limited to, m1A, m6A, I, ψ and m7G. Using SCARPET, the authors asked whether previously identified m1A and m7G sites in epitranscriptome are bona fide modifications sites or not. They show that previously reported m1A sites, contain inosine, as already reported by Wiener & Schwartz, 2021. Moreover, SCARPET is able to validate previously reported m7G sites. Overall, the manuscript is easy to read and corroborates the great potential of SCARPET as a method for orthogonally validating RNA modification mapping. In that sense, this is mostly a methodology paper with little new biology, which is fine. However, its widespread adoption may be limited, because SCARPET relies on radioactive labeling, which is increasingly discouraged. A main concern is whether SCARPET is applicable to sites with medium to low stoichiometry. In addition, some of the interpretations of the provided data seem to be overinterpretations.

Major Points

1. In Fig. 1., the authors tested the ability of SCARPET to detect modifications on sites with high stoichiometry (90-100%). However, it is not clear from the provided data that SCARPET can detect sites with lower stoichiometry. As many modification sites do not have 90-100% stoichiometry, whether SCARPET can or cannot detect such sites is a critical question. Therefore, the authors should demonstrate the limit of detection of SCARPET and if it differs from modification to modification. One way to demonstrate this could be to do a titration of synthetic RNAs with 100% modified site and mixing it with unmodified RNA, in different rations (10%, 20, 30,40%...) and monitor if they can detect modifications. Another experiment to this

end could be testing sites with lower stoichiometry from cellular RNA with SCARPET and an orthogonal approach.

Response: We thank the reviewer for raising this important point. In the current version of the manuscript, we benchmarked SCARPET primarily using high-stoichiometry rRNA sites to establish its specificity and agreement with previous LC–MS measurements. We fully agree that demonstrating SCARPET performance at lower modification stoichiometries is critical for its broader application.

First, we note that prior work has already demonstrated that SCARPET can detect sub-stoichiometric modification levels. In the preceding SCARPET study by Mirza et al. (2024), synthetic oligonucleotides were used to show sensitive detection of m^6A at defined stoichiometries, indicating that SCARPET does not require high-stoichiometry modification for robust signal. Additionally, in our present dataset SCARPET detects internal m^7G at stoichiometries as low as ~21% in cellular RNA (Fig. 4D, 4E, 4G, 4H), further supporting that the assay is compatible with lower abundance modification sites.

In response to the reviewer's suggestion, we have further strengthened the manuscript by explicitly defining the limit of SCARPET-based detection using synthetic oligonucleotides for pseudouridine and m^1A modifications. This experiment:
(i) establishes the linear relationship between input stoichiometry and the SCARPET readout and

(ii) empirically defines the accuracy and linear range of detection for different RNA modifications under our conditions.

The new data and their quantitative analysis are incorporated into the **Results** section and presented in an additional figure (Fig 2). This will clarify the practical sensitivity of SCARPET and its applicability to modification sites that are not fully modified.

2. In all SCARPET analysis done in the study, only singlet samples without replicate were provided. For confident quantification of stoichiometry and to evaluate the precision of SCARPET, the authors should include additional replicates to strengthen the reliability of the results, especially in Fig 3.

Response: We thank the reviewer for this comment. We apologize that the original manuscript was not clear about replicates. All SCARPET experiments presented in the manuscript were performed with independent biological replicates. In the original submission we showed only a single representative TLC image per condition in the main figures. We agree that explicitly presenting replicate data will improve transparency and allow a clearer assessment of the precision of SCARPET-based stoichiometry measurements.

In the revised manuscript, we have:
(i) stated in the corresponding figure legends that each experiment was performed in N = 3 (or N=2) independent replicates (as appropriate for each experiment), unless otherwise indicated.

(ii) provided the set of replicates for each experiment in the source data files.

3. The authors state that: "Based on the biochemical analysis, our data appear to resolve the m1A issue and confirm that the only sites with readily detectable m1A stoichiometry in HEK293T cells are PRUNE, ND5 and the MALAT1 ncRNA, as described previously" seems to

be an overinterpretation since only ND5 is tested by SCARPET in the study. The authors should also test PRUNE and MALAT1 with SCARPET to support this claim.

Response: We thank the reviewer for this careful and important observation. We agree that, in the unrevised version of the manuscript, only the ND5 m^1A site was directly tested by SCARPET, and that our statements regarding PRUNE1 and MALAT1 were based on previously published studies (Safra et al., 2017; Grozhik et al., 2019).

In the revised manuscript, we have conducted SCARPET on the proposed m^1A sites in endogenous PRUNE1 and MALAT1 RNAs to validate the presence of m^1A at these positions. The corresponding data is included as additional panels in Figure 5 (Panels A and B) and described in the **Results** section. These experiments directly address the reviewer's concern by providing biochemical evidence for m^1A at PRUNE1 and MALAT1 RNAs.

4. The finding that m1A sites, identified by Zhou et al., are, to a large extent, likely representing inosine is of limited novelty because Wiener& Schwart, 2021 have already demonstrated it. Adding to their finding, only three sites are validated by SCARPET in the manuscript.

Response: We thank the reviewer for raising this important point regarding the relationship between our findings and the prior analysis by Wiener & Schwartz (2021). We respectfully note that there is a fundamental distinction between what Wiener & Schwartz demonstrated and what SCARPET provides.

Wiener & Schwartz (2021) performed a computational reanalysis of the m^1A-quant-seq sequencing data from Zhou et al. and showed that many of the reported sites exhibit predominantly A-to-G mutation signatures, which are more consistent with inosine than with the A-to-T transversions expected for m^1A. This was an important bioinformatic inference; however, it did not constitute direct biochemical validation of the nucleotide identity at any of these sites, and the authors explicitly acknowledged the need for experimental confirmation. Our SCARPET analysis provides exactly this: the first direct, single-nucleotide resolution biochemical evidence confirming the presence of inosine and the unambiguous absence of m^1A at specific candidate sites.

Furthermore, the contribution of the current manuscript extends beyond the inosine finding. First, SCARPET provides direct positive biochemical validation of m^1A at the three previously established sites which computational reanalysis alone cannot achieve. Second, our comprehensive re-annotation of all candidate sites from Zhou et al., integrating A-to-I editing databases (REDIportal and DARNED), genomic variant databases (Ensembl, dbSNP), and somatic variant databases (COSMIC), provides a more complete accounting of the sources of sequence heterogeneity at these positions than was available in Wiener & Schwartz (2021), and is presented in full in Table EV1. Together, these results biochemically resolve the m^1A controversy and establish SCARPET as a generalizable tool for direct validation of RNA modification maps, a capability that bioinformatic reanalysis cannot substitute.

5. The data supporting the following statement: "Among the other m1A sites identified by Zhou et al., 19 were identified as inosine in either REDIportal or DARNED. Most of the remaining sites correspond to known genomic SNPs." is missing in the manuscript. The authors should either provide these data or delete it from the manuscript.

Response: We thank the reviewer for pointing this out. In the original submission, we summarized our re-annotation of the m^1A sites reported by Zhou et al. without including the underlying analysis. In the revised manuscript, we:

(i) have added a new figure (Figure 6C) which summarizes each of the remaining Zhou et al. candidate m^1A sites based on overlap with known genomic SNPs, SNVs or indels.

(ii) included a corresponding supplementary table (Table EV1) listing all sites, their genomic coordinates, database annotations, and classification (A-to-I editing, SNP, or other).

The sentence in the **Results** section has been revised to directly reference this new figure and table. This will ensure that our statements about re-annotation of the previously mapped m^1A sites are fully supported by the data. In addition, we have reanalyzed the Zhou et al dataset and updated the number of REDIportal-annotated inosine sites from 19 to 20 following a more comprehensive database query.

6. In Fig. 1B, and in the rest of the manuscript, a reference standard for m7G is missing. They should include the m7G reference standard in SCARPET analysis. If they are unable to do so, they should at least clearly demonstrate that in their hands, G1639 in 18S rRNA has also almost 100% stoichiometry by an orthogonal approach.

Response: We thank the reviewer for this important comment. We agree that the absence of a synthetic pm^7G standard warrants explicit explanation, and we address this in two parts. First, commercial RNA oligonucleotide synthesis companies do not offer m^7G as an internal base, and therefore a m^7G oligonucleotide standard cannot be obtained for use in SCARPET. N^7-methylation of guanosine imparts a permanent positive charge on the nucleobase, which renders the glycosidic bond inherently labile and makes m^7G-containing oligonucleotides highly susceptible to depurination (Devereaux et al., 2019; Zhang et al., 2019). Accordingly, m^7G-containing oligonucleotides undergo rapid depurination, generating abasic sites that preclude their reliable use as TLC standards.

Second, and directly addressing the reviewer's alternative suggestion, G1639 in 18S rRNA serves as a validated biological reference standard for m^7G in all SCARPET experiments. This site has been independently confirmed and benchmarked to be modified at ~98% stoichiometry by LC-MS (Taoka *et al*, 2018), and multiple other orthogonal approaches (D'Ambrosi *et al*, 2024; Zhang *et al*, 2022; Zhang *et al*, 2019). The SCARPET-measured stoichiometry of ~98% for G1639 is in excellent agreement with these established reports (Fig. 1C), demonstrating that the spot migrating above unmodified G in TLC corresponds to authentic m^7G. In the revised manuscript, we have added a statement explicitly acknowledging the chemical instability of synthetic m^7G oligonucleotides as the reason for the absence of a synthetic standard and clarifying that G1639 serves as an independently validated orthogonal reference in all m^7G SCARPET experiments.

Minor Points

1. The authors state that "The 3′-deoxyribonucleotide pairs with the nucleotide immediately upstream of the target site and directs RNase H to cleave at the 5′ side of the target residue.". However, in Fig. 1A, 5'-deoxyribonucleotide pairs with the nucleotide immediately upstream of the target site. The authors should correct this discrepancy between the figure and the text.

Response: We thank the reviewer for pointing this out. We apologize for this error in the original manuscript. The text should read: "The 5′-deoxyribonucleotide pairs with the nucleotide immediately upstream of the target site and directs RNase H to cleave at the 5′ side of the target residue." We have corrected this in the revised manuscript.

2. In Fig. 1C and all other tables displaying stoichiometry quantifications, the study from which the reference value was taken should be cited.

Response: We thank the reviewer for pointing this out. We have added the appropriate citations to all figure legends that display reference stoichiometry values. Specifically, Taoka et al. (2018) is now cited as the source of LC-MS-derived reference stoichiometry values for the rRNA modification sites shown in Figures 1C, 1G, 1J, and 1K, and other relevant legends, ensuring that the basis for each reference value is clearly attributed throughout the manuscript.

3. In Fig. 3, the authors measure the stoichiometry of previously identified m7G site by (Zhang et al., 2019) significantly lower than reported by Zhang et al. The authors should discuss this difference. Is this simply because of different labs having different batches of HEK293T that show variability on these sites, or is it because SCARPET underestimates the stoichiometry of the given sites? If possible, the authors should validate the SCARPET analysis of these sites with an orthogonal approach themselves.

Response: We thank the reviewer for raising this important point. As noted, the stoichiometries we obtain for previously reported internal m^7G sites in *EIF3A* and *RBM25* mRNAs (now Fig. 4) are lower than those reported by Zhang et al. (2019). We have explicitly discussed a plausible explanation for this difference in the revised manuscript.

The two approaches estimate stoichiometry using fundamentally different readouts. Zhang et al. inferred site occupancy from the frequency of chemically induced mutational signatures in their sequencing-based m^7G-mapping assay, an indirect measure that depends on reverse-transcription behavior, local sequence context, enrichment efficiency, and the model used to convert mutation rates into modification stoichiometry. In contrast, SCARPET directly measures the chemical identity of individual nucleotides in their native RNA context by thin-layer chromatography and quantifies the fraction of modified versus unmodified species at a given position. SCARPET has been benchmarked against LC–MS measurements on multiple rRNA sites in our study and in Mirza et al. (2024), where it shows close agreement, arguing against a systematic underestimation of stoichiometry by SCARPET.

In the revised manuscript, we have:

(i) added a paragraph in the Discussion explicitly comparing sequencing-based and SCARPET-based stoichiometry estimates, and
(ii) emphasized that, despite quantitative differences, both approaches support the key qualitative conclusion that internal m^7G occurs at appreciable stoichiometry at these sites.

4. In Fig. S2, the authors show the depletion of non-target mRNA ACTB over GAPDH mRNA. However, in the corresponding Figure legend and the text, it is not clear that the target of their enrichment is GAPDH mRNA. This creates confusion for the reader, considering that in Fig. 2, GAPDH mRNA is rather depleted as the target of BioCapT RNA enrichment in this figure is MYC mRNA. The authors should state the target mRNA in Fig. S2 more clearly.

Response: We thank the reviewer for identifying this source of confusion. We agree that the figure legend for Fig. EV2 (Fig. S2) did not clearly indicate the target mRNA for each panel, making interpretation unnecessarily difficult.

In the revised manuscript, we have updated the figure legend for Fig. EV2 to explicitly state the target mRNA for each panel. Specifically, panels A and B are now labeled to indicate that the enrichment target is *MYC* mRNA, while panel C is labeled to indicate that the enrichment target is *GAPDH* mRNA. We have also revised the corresponding text in the **Results** section to reinforce this distinction, ensuring that the reader can clearly follow which transcript is being enriched in each experiment.

5. The authors state that "... a reverse transcriptase that causes highly efficient A-to-U misincorporations upon encountering an m1A residue (Zhou et al., 2019)" appears to be scientifically incorrect. The reverse transcriptase rather causes an A-to-T mutation. Please revise accordingly.

Response: We thank the reviewer for this correction. The reviewer is absolutely right. During reverse transcription, the enzyme incorporates deoxyribonucleotides, and the signature mutation introduced at m^1A sites by the evolved reverse transcriptase is an A-to-T transversion in the cDNA sequence, not an A-to-U change. The use of "A-to-U" in the original manuscript was a scientific error. We have corrected this to "A-to-T" throughout the revised manuscript.

6. I recommend rephrasing the following sentences for clarity:
a. "To test this, we performed SCARPET on A1322, the known in 28S rRNA, which is a known m A site that is modified at nearly 100% stoichiometry"
b. "When poly(A) RNA was used, the major signal was from the adenosine spot, with a faint m 6 A signal"
c. "In this study the internal m7G sites were based on the sites that were found in common two orthogonal techniques..."

Response: We thank the reviewer for these helpful suggestions. We have rephrased all three sentences in the revised manuscript as follows:
a. The original sentence contained a garbled phrase ("the known in 28S rRNA") and redundant use of "known." It has been corrected to: "To test this, we performed SCARPET on A1322 in 28S rRNA, a known m^1A site that is modified at nearly 100% stoichiometry..."
b. The original sentence has been revised to read: "When poly(A)-enriched RNA was used, the major signal was from the adenosine spot, with only a faint m^6A signal….,".
c. The missing word "using" has been inserted and the sentence now reads: "In this study, the internal m^7G sites were based on the sites that were found in common using two orthogonal techniques, m^7G-MeRIP-seq and m^7G-seq, both applied to HeLa …."

We are deeply grateful to Referee #2 for the thorough and constructive review. The new titration experiments, the extended m^1A validation, and the comprehensive re-annotation table are all a direct result of these valuable suggestions. This manuscript is fundamentally stronger as a result of their input, and we sincerely appreciate the time and care the reviewer invested in evaluating our work.

Referee #3:

We are appreciative of Referee #3's supportive comments about the study and its contributions to resolving the m^7G and m^1A controversies. The reviewer's sole but very important concern was the absence of a p-m^7G standard in the TLC images, which we have addressed below.

RNA modifications play many important roles in gene and cell regulation. While a handful of the over 170 RNA modifications have been well characterized and established as bona fide mRNA modifications, others such as m1A and m7G have been controversial. The initial discovery of these modifications within mRNA have mostly been done by sequencing-based techniques and an orthogonal detection method has mostly (if not completely) not been utilized to validate the presence of these modifications within mRNA. Here, Nalavade et al. adapted their previous method SCARPET for m6A detection to instead detect other potential mRNA modifications like m1A, m7G, pseudouridine, m6,6A and m5C.
The results are conclusive in demonstrating that SCARPET can biochemically detect and quantify the aforementioned RNA modifications. Using SCARPET, Nalavade et al. have clearly shown that m7G is present internally within mRNA while m1A is only present in only a few mRNAs. This manuscript has provided a much-needed validation in support of m7G as an internal mRNA modification and more importantly, clarifying the m1A controversy and showing that m1A is not a widespread mRNA modification.

While not necessary for this manuscript, the authors ought to consider using SCARPET to clear up the similar controversy of ac4C being a widespread internal mRNA modification. Besides this, my only concern is that for figures 1b, 3b, 3d, 3g and S3: Where is the pm7G standard? This should be present in the TLC images. If not, is there a reason why the authors are not able to include a pm7G standard?

Response: We thank the reviewer for this thoughtful comment and suggestion.
Regarding the suggestion to apply SCARPET to the ac^4C controversy: we fully agree that the reported widespread occurrence of N^4-acetylcytidine (ac^4C) as an internal mRNA modification remains an unresolved question in the field, and that biochemical validation of the kind provided by SCARPET would be valuable. We view the application of SCARPET to ac^4C as an important and natural direction as a potential future application of the method.

Regarding the absence of a m^7G standard in the TLC images of Figs. 1B, 4A, 4D, 4G, and Fig. EV3: Commercial oligonucleotide synthesis suppliers do not offer synthetic oligonucleotides with m^7G due to the instability of m^7G. N^7-methylation of guanosine imparts a permanent positive charge on the nucleobase, which makes m^7G-containing oligonucleotides highly susceptible to depurination (Devereaux *et al*, 2019). We have added a statement to the revised manuscript explaining the chemical basis for the absence of a synthetic m^7G standard and clarifying our rationale for the use of G1639 in the 18S rRNA as the validated reference.

We are very grateful to Referee #3 for the enthusiastic and supportive assessment of our work. We especially appreciate the thoughtful suggestion to apply SCARPET to the ac^4C controversy, which we view as an exciting and natural future direction for the method. We thank the reviewer for encouragement and for recognizing the broader utility of SCARPET for the field.

We would like to sincerely thank all the reviewers for their time and expertise to provide such thoughtful and constructive feedback on our manuscript. Their suggestions have led to important new experiments and analyses that have meaningfully improved the rigor and scope of the study. We are grateful for their engagement with our work, and we hope that with these changes, the reviewers will find the manuscript acceptable for publication in *EMBO Reports*.

References:

D'Ambrosi S, Raquel G-V, Darek K, Patrice V, Nuria M-C, Laura B, Monika G-L, M. AA, Sabine D, Antonio H *et al* (2024) Global and single-nucleotide resolution detection of 7-methylguanosine in RNA. *RNA Biology* 21: 476–493

Devereaux ZJ, Zhu Y, Rodgers M (2019) Relative glycosidic bond stabilities of naturally occurring methylguanosines: 7-methylation is intrinsically activating. *European Journal of Mass Spectrometry* 25: 16–29

Taoka M, Nobe Y, Yamaki Y, Sato K, Ishikawa H, Izumikawa K, Yamauchi Y, Hirota K, Nakayama H, Takahashi N *et al* (2018) Landscape of the complete RNA chemical modifications in the human 80S ribosome. *Nucleic Acids Research* 46: 9289–9298

Zhang L-S, Ju C-W, Liu C, Wei J, Dai Q, Chen L, Ye C, He C (2022) m7G-quant-seq: Quantitative Detection of RNA Internal N7-Methylguanosine. *ACS Chemical Biology* 17: 3306–3312

Zhang L-S, Liu C, Ma H, Dai Q, Sun H-L, Luo G, Zhang Z, Zhang L, Hu L, Dong X *et al* (2019) Transcriptome-wide Mapping of Internal N7-Methylguanosine Methylome in Mammalian mRNA. *Molecular Cell* 74: 1304–1316.e1308

Dear Samie,

Thank you for the submission of your revised manuscript. We have now received the enclosed reports from the referees and I am happy to say that both support its publication now. Only a few editorial requests will need to be addressed before we can proceed with the official acceptance of your manuscript:

- Please delete these sentences from the DAS: "The data supporting the findings of this study are available in the source data file of the article and Table EV1. No new sequencing datasets were generated in this study."

- The author credits need to be removed from the ms file. All credits need to be entered during online ms submission.

- In the author checklist, please fill out all questions on statistics and send us a new, completed checklist.

- The FUNDING INFO T32 CA062948 is missing in our online submission system, please add. The funding info in the ms needs to be provided under Acknowledgments and no separate Funding section heading is needed.

- The table callouts in the Reagents table need to be updated to Dataset EV1 and Dataset EV2.

- Table EV1 and Table EV2 are Datasets and need to be updated to Dataset EV1 and Dataset EV2 in all places: source file names, titles in the system, callouts in the ms.

- The Reagents & Tools TABLE needs to be removed from the ms file and uploaded as a separate file.

- The Source Data (SD) needs to be uploaded as one folder per main figure. The SD for EV Figures can be combined into one folder.

* Figure Legends - Comments *

- Please note that the exact p values are not provided in the legends of figures 3B,C,D; 4C,F; EV-2A,B,C. Please provide exact values as reasonable.
- Please indicate the statistical test used for data analysis in the legends of figures 3B,C,D.
- Please note that information related to n is missing in the legend of 3D.
- Although 'n' is provided, please describe the nature of entity for 'n' in the legends of figures 3B,C; EV-2A,B,C.
- Please note that the error bars are not defined in the legends of figures 3B,C,D; EV-2A,B,C.

EMBO press papers are accompanied online by A) a short (1-2 sentences) summary of the findings and their significance, B) 2-3 bullet points highlighting key results and C) a synopsis image that is exactly 550 pixels wide and 300 pixels high. The synopsis image should provide a sketch of the major findings, like a graphical abstract. Please note that text needs to be readable at the final size. Please send us this information along with the final manuscript.

I look forward to seeing a final version of your manuscript as soon as possible.

Kind regards,
Esther

Esther Schnapp, PhD
Senior Editor
EMBO reports

Referee #1:

The authors have addressed the concerns I raised in the revision.

Referee #2:

The authors have adequately revised the ms from our end.

All minor editorial requests have been addressed by the authors.

2nd Revision - Editorial Decision **8th Jun 2026**

Prof. Samie Jaffrey
Weill Medical College, Cornell University
Pharmacology
1300 York Ave., Box 70
Box 70
New York, NY 10065
United States

Dear Samie,

I am very pleased to accept your manuscript for publication in the next available issue of EMBO reports. Thank you for your contribution to our journal.

Your manuscript will be processed for publication by EMBO Press. It will be copy edited and you will receive page proofs prior to publication. Please note that you will be contacted by Springer Nature Author Services to complete licensing and payment information.

You may qualify for financial assistance for your publication charges - either via a Springer Nature fully open access agreement or an EMBO initiative. Check your eligibility: https://link.springer.com/journal/44319/how-to-publish-with-us

Should you be planning a Press Release on your article, please notify the production team via this form in order to coordinate publication and release dates: https://forms.cloud.microsoft/e/eH4A8JVP6g

If you have any questions, please do not hesitate to contact the Editorial Office. Thank you for your contribution to EMBO Reports.

Best regards,
Esther

Esther Schnapp, PhD
Senior Editor
EMBO reports

>>> Please note that it is EMBO Reports policy for the transcript of the editorial process (containing referee reports and your response letter) to be published as an online supplement to each paper. If you do NOT want this, you will need to inform the Editorial Office via email immediately. More information is available here: https://link.springer.com/partners/embo-press/editorial-policies#Peer%20review

EMBO Press Author Checklist

Corresponding Author Name: Samie R. Jaffrey	
Journal Submitted to: EMBO Reports	
Manuscript Number: EMBOR-2025-63274V2	

USEFUL LINKS FOR COMPLETING THIS FORM
The EMBO Journal - Author Guidelines
EMBO Reports - Author Guidelines
Molecular Systems Biology - Author Guidelines
EMBO Molecular Medicine - Author Guidelines

Reporting Checklist for Life Science Articles (updated January
This checklist is adapted from Materials Design Analysis Reporting (MDAR) Checklist for Authors. MDAR establishes a minimum set of requirements in transparent reporting in the life sciences (see Statement of Task: 10.31222/osf.io/9sm4x). Please follow the journal's guidelines in preparing your
Please note that a copy of this checklist will be published alongside your article.

Abridged guidelines for figures
1. Data
The data shown in figures should satisfy the following conditions:
- the data were obtained and processed according to the field's best practice and are presented to reflect the results of the experiments in an accurate and unbiased manner.
- ideally, figure panels should include only measurements that are directly comparable to each other and obtained with the same assay.
- plots include clearly labeled error bars for independent experiments and sample sizes. Unless justified, error bars should not be shown for technical
- if n<5, the individual data points from each experiment should be plotted. Any statistical test employed should be justified.
- Source Data should be included to report the data underlying figures according to the guidelines set out in the authorship guidelines on Data

2. Captions
Each figure caption should contain the following information, for each panel where they are relevant:
- a specification of the experimental system investigated (eg cell line, species name).
- the assay(s) and method(s) used to carry out the reported observations and measurements.
- an explicit mention of the biological and chemical entity(ies) that are being measured.
- an explicit mention of the biological and chemical entity(ies) that are altered/varied/perturbed in a controlled manner.
- the exact sample size (n) for each experimental group/condition, given as a number, not a range;
- a description of the sample collection allowing the reader to understand whether the samples represent technical or biological replicates (including how many animals, litters, cultures, etc.).
- a statement of how many times the experiment shown was independently replicated in the laboratory.
- definitions of statistical methods and measures:
  - common tests, such as t-test (please specify whether paired vs. unpaired), simple χ2 tests, Wilcoxon and Mann-Whitney tests, can be unambiguously identified by name only, but more complex techniques should be described in the methods section;
  - are tests one-sided or two-sided?
  - are there adjustments for multiple comparisons?
  - exact statistical test results, e.g., P values = x but not P values < x;
  - definition of 'center values' as median or average;
  - definition of error bars as s.d. or s.e.m.

Please complete ALL of the questions below.
Select "Not Applicable" only when the requested information is not relevant for your study.

Materials

Newly Created Materials	Information included in the manuscript?	In which section is the information available? (Reagents and Tools Table, Materials and Methods, Figures, Data Availability Section)
New materials and reagents need to be available; do any restrictions apply?	Not Applicable	

Antibodies	Information included in the manuscript?	In which section is the information available? (Reagents and Tools Table, Materials and Methods, Figures, Data Availability Section)
For **antibodies** provide the following information:<br>- Commercial antibodies: RRID (if possible) or supplier name, catalogue number and or/clone number<br>- Non-commercial: RRID or citation	Not Applicable	

DNA and RNA sequences	Information included in the manuscript?	In which section is the information available? (Reagents and Tools Table, Materials and Methods, Figures, Data Availability Section)
**Short novel DNA or RNA including primers, probes**: provide the sequences.	Yes	Table EV2

Cell materials	Information included in the manuscript?	In which section is the information available? (Reagents and Tools Table, Materials and Methods, Figures, Data Availability Section)
**Cell lines:** Provide species information, strain. Provide accession number in repository **OR** supplier name, catalog number, clone number, and/**OR** RRID.	Yes	Materials and Methods
**Primary cultures:** Provide species, strain, sex of origin, genetic modification status.	Not Applicable	
Report if the cell lines were recently **authenticated** (e.g., by STR profiling) and tested for mycoplasma contamination.	Not Applicable	

Experimental animals	Information included in the manuscript?	In which section is the information available? (Reagents and Tools Table, Materials and Methods, Figures, Data Availability Section)
**Laboratory animals or Model organisms:** Provide species, strain, sex, age, genetic modification status. Provide accession number in repository **OR** supplier name, catalog number, clone number, **OR** RRID.	Not Applicable	
**Animal observed in or captured from the field:** Provide species, sex, and age where possible.	Not Applicable	
Please detail **housing and husbandry conditions**.	Not Applicable	

Plants and microbes	Information included in the manuscript?	In which section is the information available? (Reagents and Tools Table, Materials and Methods, Figures, Data Availability Section)
**Plants:** provide species and strain, ecotype and cultivar where relevant, unique accession number if available, and source (including location for collected wild specimens).	Not Applicable	
**Microbes:** provide species and strain, unique accession number if available, and source.	Not Applicable	

Human research participants	Information included in the manuscript?	In which section is the information available? (Reagents and Tools Table, Materials and Methods, Figures, Data Availability Section)
If collected and within the bounds of privacy constraints report on age, sex and gender or ethnicity for all study participants.	Not Applicable	

Core facilities	Information included in the manuscript?	In which section is the information available? (Reagents and Tools Table, Materials and Methods, Figures, Data Availability Section)
If your work benefited from core facilities, was their service mentioned in the acknowledgments section?	Not Applicable	

Design

Study protocol	Information included in the manuscript?	In which section is the information available? (Reagents and Tools Table, Materials and Methods, Figures, Data Availability Section)
If study protocol has been **pre-registered, provide DOI in the manuscript**. For clinical trials, provide the trial registration number **OR** cite DOI.	Not Applicable	
Report the **clinical trial registration number** (at ClinicalTrials.gov or equivalent), where applicable.	Not Applicable	

Laboratory protocol	Information included in the manuscript?	In which section is the information available? (Reagents and Tools Table, Materials and Methods, Figures, Data Availability Section)
Provide DOI OR other citation details if **external detailed step-by-step protocols** are available.	Not Applicable	

Experimental study design and statistics	Information included in the manuscript?	In which section is the information available? (Reagents and Tools Table, Materials and Methods, Figures, Data Availability Section)
Include a statement about **sample size** estimate even if no statistical methods were used.	Yes	Figure Legends
Were any steps taken to minimize the effects of subjective bias when allocating animals/samples to treatment (e.g. **randomization procedure**)? If yes, have they been described?	Not Applicable	
Include a statement about **blinding** even if no blinding was done.	Not Applicable	
Describe **inclusion/exclusion criteria** if samples or animals were excluded from the analysis. Were the criteria pre-established?<br><br>If sample or data points were omitted from analysis, report if this was due to attrition or intentional exclusion and provide justification.	Yes	Figure Legends
For every figure, are **statistical tests** justified as appropriate? Do the data meet the assumptions of the tests (e.g., normal distribution)? Describe any methods used to assess it. Is there an estimate of variation within each group of data? Is the variance similar between the groups that are being statistically compared?	Yes	Figure Legends

Sample definition and in-laboratory replication	Information included in the manuscript?	In which section is the information available? (Reagents and Tools Table, Materials and Methods, Figures, Data Availability Section)
In the figure legends: state number of times the experiment was **replicated** in laboratory.	Yes	Figure legends
In the figure legends: define whether data describe **technical or biological replicates**.	Yes	Figure legends

Ethics

Ethics	Information included in the manuscript?	In which section is the information available? (Reagents and Tools Table, Materials and Methods, Figures, Data Availability Section)
Studies involving **human participants**: State details of **authority granting ethics approval** (IRB or equivalent committee(s), provide reference number for approval.	Not Applicable	
Studies involving **human participants**: Include a statement confirming that **informed consent** was obtained from all subjects and that the experiments conformed to the principles set out in the WMA Declaration of Helsinki and the Department of Health and Human Services Belmont Report.	Not Applicable	
Studies involving **human participants:** For publication of **patient photos**, include a statement confirming that consent to publish was obtained.	Not Applicable	
Studies involving experimental **animals**: State details of **authority granting ethics approval** (IRB or equivalent committee(s), provide reference number for approval. Include a statement of compliance with ethical regulations.	Not Applicable	
Studies involving **specimen and field samples:** State if relevant **permits** obtained, provide details of authority approving study; if none were required, explain why.	Not Applicable	

Dual Use Research of Concern (DURC)	Information included in the manuscript?	In which section is the information available? (Reagents and Tools Table, Materials and Methods, Figures, Data Availability Section)
Could your study fall under dual use research restrictions? Please check biosecurity documents and list of **select agents and toxins** (CDC): https://www.selectagents.gov/sat/list.htm	Not Applicable	
If you used a select agent, is the security level of the lab appropriate and reported in the manuscript?	Not Applicable	
If a study is subject to dual use research of concern regulations, is the name of the **authority granting approval and reference number** for the regulatory approval provided in the manuscript?	Not Applicable	

Reporting

The MDAR framework recommends adoption of discipline-specific guidelines, established and endorsed through community initiatives. Journals have their own policy about requiring specific guidelines and recommendations to complement MDAR.

Adherence to community standards	Information included in the manuscript?	In which section is the information available? (Reagents and Tools Table, Materials and Methods, Figures, Data Availability Section)
State if relevant guidelines or checklists (e.g., **ICMJE, MIBBI, ARRIVE, PRISMA**) have been followed or provided.	Not Applicable	
For **tumor marker prognostic studies,** we recommend that you follow the **REMARK** reporting guidelines (see link list at top right). See author guidelines, under 'Reporting Guidelines'. Please confirm you have followed these guidelines.	Not Applicable	
For **phase II and III randomized controlled trials**, please refer to the **CONSORT** flow diagram (see link list at top right) and submit the CONSORT checklist (see link list at top right) with your submission. See author guidelines, under 'Reporting Guidelines'. Please confirm you have submitted this list.	Not Applicable	

Data Availability

Data availability	Information included in the manuscript?	In which section is the information available? (Reagents and Tools Table, Materials and Methods, Figures, Data Availability Section)
Have **primary datasets** been deposited according to the journal's guidelines (see 'Data Deposition' section) and the respective accession numbers provided in the Data Availability Section?	Not Applicable	
Were **human clinical and genomic datasets** deposited in a public access-controlled repository in accordance to ethical obligations to the patients and to the applicable consent agreement?	Yes	Data Availability
Are **computational models** that are central and integral to a study available without restrictions in a machine-readable form? Were the relevant accession numbers or links provided?	Yes	References
If publicly available data were reused, provide the respective **data citations in the reference list**.	Yes	References
